# Nitrogen speciation and transformations in fire-derived organic matter

**DOI:** 10.1016/j.gca.2020.02.034

**Published:** 2020-05-01

**Authors:** Dorisel Torres-Rojas, Rachel Hestrin, Dawit Solomon, Adam W. Gillespie, James J. Dynes, Tom Z. Regier, Johannes Lehmann

**Affiliations:** a Soil and Crop Sciences, Cornell University, Ithaca, NY 14853, USA; b School of Environmental Sciences, University of Guelph, Guelph, ON, Canada; c Canadian Light Source Inc., Saskatoon, SK, Canada; d CGIAR Research Program on Climate Change, Agriculture and Food Security (CCAFS), P.O. Box 5689, Addis Ababa, Ethiopia; e Atkinson Center for a Sustainable Future, Cornell University, Ithaca, NY 14853, USA

**Keywords:** Fire, Pyrogenic organic N, N content, NEXAFS, Aromatic N heterocycles, Organic C persistence, Biochar

## Abstract

Vegetation fires are known to have broad geochemical effects on carbon (C) cycles in the Earth system, yet limited information is available for nitrogen (N). In this study, we evaluated how charring organic matter (OM) to pyrogenic OM (PyOM) altered the N molecular structure and affected subsequent C and N mineralization. Nitrogen near-edge X-ray absorption fine structure (NEXAFS) of uncharred OM, PyOM, PyOM toluene extract, and PyOM after toluene extraction were used to predict PyOM-C and -N mineralization potentials. PyOM was produced from three different plants (e.g. Maize-*Zea mays* L.; Ryegrass-*Lollium perenne* L.; and Willow-*Salix viminalix* L.) each with varying initial N contents at three pyrolysis temperatures (350, 500 and 700 °C). Mineralization of C and N was measured from incubations of uncharred OM and PyOM in a sand matrix for 256 days at 30 °C. As pyrolysis temperature increased from 350 to 700 °C, aromatic C=N in 6-membered rings (putative) increased threefold. Aromatic C=N in 6-membered oxygenated ring increased sevenfold, and quaternary aromatic N doubled. Initial uncharred OM-N content was positively correlated with the proportion of heterocyclic aromatic N in PyOM (R^2^ = 0.44*; P* < 0.0001; n = 42). A 55% increase of aromatic N heterocycles at high OM-N content, when compared to low OM-N content, suggests that higher concentrations of N favor the incorporation of N atoms into aromatic structures by overcoming the energy barrier associated with the electronic and atomic configuration of the C structure. A ten-fold increase of aromatic C=N in 6-membered rings (putative) in PyOM (as proportion of all PyOM-N) decreased C mineralization by 87%, whereas total N contents and C:N ratios of PyOM had no effects on C mineralization of PyOM-C for both pyrolysis temperatures (for PyOM-350 °C, R^2^ = 0.15; *P* < 0.27; for PyOM-700 °C, R^2^ = 0.22; *P* < 0.21). Oxidized aromatic N in PyOM toluene extracts correlated with higher C mineralization, whereas aromatic N in 6-membered heterocycles correlated with reduced C mineralization (R^2^ = 0.56; *P =* 0.001; n = 100). Similarly, aromatic N in 6-membered heterocycles in PyOM remaining after toluene extraction reduced PyOM-C mineralization (R^2^ = 0.49; *P* = 0.0006; n = 100). PyOM-C mineralization increased when N atoms were located at the edge of the C network in the form of oxidized N functionalities or when more N was found in PyOM toluene extracts and was more accessible to microbial oxidation. These results confirm the hypothesis that C persistence of fire-derived OM is significantly affected by its molecular N structure and the presented quantitative structure-activity relationship can be utilized for predictive modeling purposes.

## 1 Introduction

Vegetation fires are part of Earth systems and significantly influence global carbon (C) cycles and geochemistry (Bowman et al., 2009). As a response to climate change, fire frequency, and severity have recently increased (Wotton et al., 2017). Fires produce copious amounts of CO_2_ and yield a significant proportion of solid char residues, so-called pyrogenic organic matter (PyOM) (Schmidt and Noack, 2000, Preston and Schmidt, 2006, Hilscher and Knicker, 2011). PyOM is recognized as an important and ubiquitous pool of global C found in the atmosphere, soils, rivers, lakes, ocean waters and sediments (Bird et al., 2015, Reisser et al., 2016, Santín et al., 2016). PyOM forms a continuum of slightly charred to highly transformed organic matter, which is described as clusters of polyaromatic rings with nitrogen (N), oxygen (O) and sulfur (S) substitution (Knicker, 2010). The molecular changes of OM-C during charring (Czimczik et al., 2002, Keiluweit et al., 2010, Heymann et al., 2011, Wiedemeier et al., 2015) and the relationship to its biological mineralization have been extensively studied (Baldock and Smernik, 2002, Nguyen et al., 2010). However, N in PyOM and how it is related to the persistence of PyOM-C has received much less attention.

The composition and structure of PyOM are some of the main drivers for the persistence of PyOM-C in the environment (Bird et al., 2015). During thermal degradation, the degree of aromaticity and condensation of PyOM-C increases (McBeath et al., 2011, McBeath et al., 2015, Wiedemeier et al., 2015). Likewise, N in organic matter (OM-N) is structurally altered and incorporated into the aromatic structure of PyOM as thermal degradation proceeds, producing aromatic N heterocycles in PyOM-N (Knicker et al., 1996, Almendros et al., 2003, Knicker, 2007, Hilscher et al., 2009, Chen et al., 2014, Pyle et al., 2015). It is unclear how the initial N content of uncharred OM influences the formation of aromatic N structures in PyOM, beyond the C and N stoichiometry. Several authors (Stańczyk et al., 1995, Almendros et al., 2003) have identified the formation mechanisms of aromatic N heterocycles during the thermal degradation of OM. Stańczyk et al. (1995) observed increasing N contents in PyOM when the N precursor was an aromatic ring structure. They suggested that aromatic N structures are more thermally stable when compared to non-aromatic N structures. Almendros et al. (2003) proposed the formation of new aromatic N structures during thermal degradation but did not report on the effects of N amounts on any pathways of N incorporation into PyOM.

Additionally a few studies (dos Santos and Alvarez, 1998, Chen et al., 2010) have reported that the formation of aromatic N structures in N-doped carbon nanotubes are affected by the amount of initial N added. They suggested that there is a relationship between the amount of exogenous N added to the precursor and the energy barrier for the formation of different N moieties. These findings may suggest that with low initial N contents of the OM itself, non-aromatic N structures will be preferentially formed during pyrolysis; however, as N content increases, a greater proportion of aromatic N structures may form. To our knowledge, evaluating the effect of varying N concentration of the original OM on the formation of PyOM with higher non-aromatic N structures has not been directly shown.

In the last decade, several studies have shown evidence for the persistence of PyOM in soils (Lehmann et al., 2015, Wang et al., 2016). The extent of its mineralization depends on the temperatures during fires and the resulting composition and structure of PyOM-C, particularly the degree of aromatic condensation (McBeath et al., 2011, Singh et al., 2012, Wiedemeier et al., 2015). However, PyOM is a heterogeneous material that includes various forms of aromatic C structures. The connection between persistence and C composition of PyOM has been extensively studied but there is less understanding of N functional composition and its effect on C mineralization. Similar to C, the aromatic N structures in PyOM vary widely with the degree of thermal degradation (Knicker, 2007) and may, therefore, affect the mineralization of PyOM-C. For OM, conventional wisdom suggests that N contents and C:N ratios drive C mineralization (Melillo et al., 1982). While the quantitative relationship between N functionalities in plant residues (e.g., lignin:N ratios) and C decomposition is well studied (Fox et al., 1990), it is not known how changes in N functional group composition in PyOM as a function of different N contents in uncharred OM may affect the mineralization of PyOM-C structures.

PyOM contains weakly aromatic or non-aromatic C fractions that are susceptible to microbial degradation (Lehmann et al., 2015). Similarly, several studies (Hilscher et al., 2009, Hilscher and Knicker, 2011, de la Rosa and Knicker, 2011, Wang et al., 2012b) have shown evidence of biological degradation of PyOM-N. According to these studies, microbes can access some forms of PyOM-N that are used for microbial biomass production and eventual mineralization. These results suggest that a fraction of the PyOM-N is easily mineralizable. This fraction may also have a direct impact on the mineralization of the associated PyOM-C. However, it is not clear which physical fraction of PyOM-N is biologically available, what proportion it constitutes as a result of different N contents in the uncharred OM, and whether and in what way these PyOM-N functionalities affect the mineralization of PyOM-C.

This study focused on the molecular changes of N during thermal degradation of plant material as a function of initial N contents and its subsequent effect on the biological degradation of PyOM-C. The objectives were (1) to determine the effects of varying N contents of uncharred plant OM and increased charring temperature on the functional group composition of N in the resulting PyOM; and (2) to evaluate how the content and molecular structure of N in PyOM affected the mineralization of PyOM-C. We hypothesized that (i) higher N concentrations in uncharred plant material increase the proportion of aromatic N heterocycles in PyOM-N; and (ii) there is a fraction of PyOM with an N molecular structure that is easily mineralizable and favors PyOM-C mineralization.

## 2 Materials and methods

### 2.1 Plant residues and PyOM production

Biomass burning under ideal combustion conditions (stoichiometric oxygen) produces water, carbon dioxide (CO_2_) and ash. However, in nature, these conditions are rarely found, and the products of incomplete combustion include PyOM. The PyOM production parameters for this study include different vegetation types and temperatures potentially found during a vegetation fires (Neary et al., 1999, Schmidt and Noack, 2000, González-Pérez et al., 2004, Knicker, 2007, Santin et al., 2013). Vegetation fires include a wide variety of conditions that we were not able to cover experimentally, and the plant residues, heating conditions and temperatures do not cover the entire range of possible conditions.

We used plant residues of corn-*Zea mays* L*.*; ryegrass-*Lollium perenne* L*.*; and willow-*Salix viminalix* L*.* with varying N content (see Supplementary Section 1.1). Plant residues were separated into leaves, stalks and woody residues, to obtain plant tissue with different N contents. The residues were dried at 60 °C to a constant mass and ground to ≤2 mm. We produced PyOM under a set of varying parameters that include environmental conditions found during natural fires. Approximately 15 g of plant residues were placed in a custom-built batch reactor and purged under Ar gas. The plant residues were heated at a rate of 2.5 °C min^−1^ and allowed to remain for 30 min at the highest heat temperatures (HHT) of 350, 500 and 700 °C. The reactor cooled down to 25 °C at a rate of 5 °C min^−1^. The resulting PyOM was ground and sieved to 74–150 μm.

We measured total C and N for uncharred OM and PyOM on ground samples (<74 μm) (Supplementary Table 1) on an NC2500 (Carlo Erba, Italy) elemental analyzer coupled to a Delta V Isotope Ratio Mass Spectrometer (Thermo Scientific, Germany). The inorganic N contents for uncharred OM and PyOM were measured by mixing 0.5 g of the sample with 30 mL of 1 M KCl and shaking for one hour. The KCl extract was filtered through a Whatman No. 42 filter paper. The concentrations of ammonium (NH_4_^+^) and nitrate (NO_3_^−^) in solution were determined by continuous flow analysis (Bran and Luebbe Autoanalyzer, SPX, Charlotte, NC, USA). PyOM pH was measured in double distilled and deionized water (DDIW) at a ratio of 1:20 g mL^−1^ using a glass electrode (Orion 3-Star pH Benchtop; Thermo Electron Corporation, Beverly, MA, USA) (Supplementary Table 2).

### 2.2 PyOM extraction and fractionation

The heating of OM during vegetation fires produces organic substances some of which volatilize, and others that condense at the litter-soil interface (Savage et al., 1972, Savage, 1974, DeBano, 2000, Knicker, 2007) where PyOM remains immediately after vegetation fires. Savage et al. (1972) suggested that these substances are products of the pyrolysis process of organic matter. We extracted PyOM using toluene to generate a residual PyOM after toluene extraction and a PyOM toluene extract. PyOM after toluene extraction represents the toluene-insoluble solid material remaining after the extraction. The PyOM toluene extract represents organic compounds that are likely re-condensed on PyOM surfaces and pores during vegetation fires, they are hydrophobic and therefore extractable by a non-polar substance such as toluene (Cole et al., 2012).

We extracted PyOM with toluene (HPLC grade; Sigma-Aldrich, USA) in a Soxhlet apparatus (Jonker and Koelmans, 2002). This fraction of PyOM is considered the toluene-extractable fraction produced during pyrolysis. A subsample of 100 mg of PyOM, was weighed into a glass extraction thimble with fritted disc (Wilmad Lab Glass, Vineland, NJ, USA), treated with 70 mL of toluene, which recirculated through the apparatus for 2 h. The PyOM toluene extract was cooled down and filtered with Whatman no. 42 paper (Whatman International Ltd, England). The extract was further concentrated using a rotary evaporator (Rotavapor R-134, Büchi Labortechnik AG, Switzerland). PyOM after toluene extraction was dried at 70 °C to constant weight. Both materials were stored for further analysis.

### 2.3 NEXAFS

The N chemical speciation of uncharred plant OM, PyOM, PyOM after toluene extraction and the PyOM toluene extract was obtained using N (1s) K near edge X-ray adsorption fine structure (NEXAFS) spectroscopy. Samples (see Supplementary Section 1.2 for sample preparation) were mounted on Au coated Si wafers and spectra were acquired using the slew scanning mode on the 11ID-1 spherical grating monochromator (SGM) beamline at the Canadian Light Source (CLS, Saskatoon, Saskatchewan, Canada). The beamline is capable of providing 10^11^ photons s^−1^ at the N K-edge with a resolving power (E/ΔE) better than 10,000 (Regier et al., 2007a, Regier et al., 2007b). At the time of collection, ring current was filled to 250 mA every 8 h. Solid samples were scanned ten times at random locations on the sample. Due to the lower N concentration of the PyOM toluene extract, 20 scans were taken at separate sites on the sample to improve the signal to noise ratio. Each scan took 20 s and beam spot size was 1000 μm by 100 μm (Regier et al., 2007a, Regier et al., 2007b). The exit slit gap was set at 25 μm and the photon energy was scanned from 390 to 420 eV. Total electron yield (TEY) was acquired by monitoring the sample drain current. The incident photon flux on the sample (I_0_) was recorded in subsequent scans using a Si photodiode (IRD). The photon energy was calibrated to the N 1s → π* υ = 0 vibronic for N_2_ gas at 400.80 eV using ammonium sulfate (Gillespie et al., 2008).

Radiation damage was investigated by irradiating uncharred OM samples multiple times at the same sample location. If the OM experienced radiation damage we would expect to see new and intense spectral features with increased radiation exposure. Uncharred OM irradiated at different locations throughout the sample did not show new spectral features (Supplementary Fig. 1a). When OM was irradiated at the same position multiple times (Supplementary Fig. 1b), a new spectral feature developed at 398.78 eV, most likely a product of amino acid or peptide decomposition by soft X-rays (Leinweber et al., 2007). Therefore, we do not expect our method of scanning at different random locations to cause radiation damage.

Nitrogen spectra were processed using IGOR Pro (Wavemetrics, Oregon, USA) and Athena (Ravel and Newville, 2005) software packages. The incident beam intensity (I_0_) and sample measurements were scaled and offset using customized macros in IGOR Pro (Gillespie et al., 2014a, Gillespie et al., 2014b). Normalized spectra were obtained by dividing the sample intensity by the I_0_. The data were averaged for five scans and background corrected by a linear regression fit through the pre-edge and post-edge regions and normalized to an edge jump of 1.

The N K-edge of all sample spectra were deconvoluted using a non-linear least-square fitting method (see Supplementary Section 1.3 for details on deconvolution process). The analysis process does not yield quantitative N functionality values; instead it provides a numerical approach for evaluation of differences in spectral features of samples of similar overall concentrations. Deconvolution was performed using an arctangent function to model the edge step and fixed at 404 eV. Spectral features were resolved using a series of Gaussian peaks representing the main N 1s → π*/1s → σ* transitions (Fityk 0.9.8, Wojdyr, 2010) (see Supplementary Figs. 2–3 and Supplementary Table 3 for N standard spectra and peak position and Supplementary Table 4 for peak assignments used in deconvolution). The proportion of the 1s → π* area (as a fraction of the entire spectral area) for N bonds was calculated for each sample (Supplementary Tables 5–9).

### 2.4 OM and PyOM incubations

Incubation experiments were used to determine uncharred OM- and PyOM-C and -N mineralization. For C mineralization, a mixture of 200 mg of uncharred OM or PyOM and 15 g of ashed quartz sand (550 °C for 2 hrs) (Sigma Aldrich no. 274739, 50–70 mesh) were mixed and added to 60-mL airtight jars. For each uncharred OM or PyOM-sand mixture, the water holding capacity (WHC) was determined gravimetrically (Haney and Haney, 2010). CO_2_-free water and microbial inoculum were added to each sample jar to achieve 55% WHC.

In brief, the microbial inoculum was isolated from historical charcoal furnace soil samples from Alabama (Cheng et al., 2008). A homogenous mixture of surface, subsurface soil and nutrient solution was incubated at 30 °C and 55% WHC for seven days to stimulate microbial activity. The soil–water mixture was filtered through a Whatman #1 filter paper to isolate the microbial inoculum. To obtain the final microbial inoculum nutrient solution without N (Cheng et al., 2008) was added to the inoculum and incubated again at 30 °C for another seven days.

Individual sample jars were placed inside a 473-mL Mason jar with a vial containing 15 mL of 0.09 M KOH. CO_2_-free distilled deionized water (DDIW) (5 mL) was added to the bottom of the jar to keep the environment moist (Whitman et al., 2014). Each treatment had four replicates, and an additional four blanks with no uncharred OM or PyOM additions were included and incubated at 30 °C for 252 days. CO_2_ trapped in the KOH solution during the incubation was quantified by measuring the electrical conductivity (EC) of the solution. Due to differences in CO_2_ respiration rates between uncharred OM and PyOM, EC measurements were taken at different time intervals. For uncharred OM, the EC of the KOH traps was measured on days 7, 14, 21, 42, 113, 252. Fresh KOH traps were immediately replaced in the Mason jars, and 5 mL of fresh CO_2_-free DDIW were added to the bottom of the jar. The EC of KOH traps for PyOM-350 °C and PyOM-700 °C were measured on days 7, 21, 49, 113, 252 and 7, 21, 63, 186 and 252, respectively. For rapid and higher result consistency, total C mineralized was determined by correlating the measured EC to a known volume of CO_2,_ using a calibration curve (Strotmann et al., 2004, Woo et al., 2016).

Nitrogen mineralization was determined by measuring the amount of available mineral N at the beginning and end of the incubation period. At time 0, mineralized N was determined immediately after additions of nutrient solution and microbial inoculum. For incubated sample jars (t = 50 days), 200 mg of OM or PyOM were mixed with 15 g of ashed quartz sand (after exposure to 550 °C for 2 hrs to remove any remaining organic C). Inoculum and additional DDIW was added to bring each jar to 55% WHC. Sample jars were incubated at 30 °C for a period of 50 days. The blank treatment consisted of sand without uncharred OM or PyOM to which 2.16 mL of nutrient solution and microbial inoculum were added. For all samples, mineralized inorganic N (NH_4_-N + NO_3_-N) was extracted by adding 2 M KCl in a 1:2 w/v ratio. All sample-KCl mixtures were shaken for 1 h after which the supernatant was filtered (Whatman no. 42) and the filtrate analyzed for NH_4_-N and NO_3_-N on an autoanalyzer (Bran and Luebbe Autoanalyzer, SPX, Charlotte, NC, USA). Net N mineralization was calculated as the difference between the NH_4_-N and NO_3_-N concentrations in the incubated sample (t = 50 days) and the sample at t = 0 days after the blank was subtracted.

### 2.5 Statistical analysis

All statistical analyses were performed using JMP version 12.0.1 (SAS Institute, 2015). We used linear regression models (*P* ≤ 0.05) to fit the proportion of aromatic (sum of all aromatic heterocycles including 5 and 6-membered rings) and non-aromatic N in PyOM with the N content of PyOM. We also fitted a linear regression to the relationship between cumulative C mineralization of either OM or PyOM to the respective C:N ratios.

Multivariate regression analysis was used to determine the relationship between cumulative PyOM-C mineralized and proportions of N functional groups (as a function of the sum of all N functional groups) from the different fractions of PyOM as quantified by N K-edge NEXAFS. Stepwise regression was performed to select the most significant of the independent variables for inclusion in multivariate linear models for the dependent variables. *P*-value thresholds of 0.1 were used to allow an independent variable to enter the model, whereas 0.05 was used to enable an independent variable to be removed from the model. A mixed stopping rule was applied to allow the alternation of forward and backward steps.

## 3 Results

### 3.1 Nitrogen content changes with temperature and initial N content

**Fig. 1:**
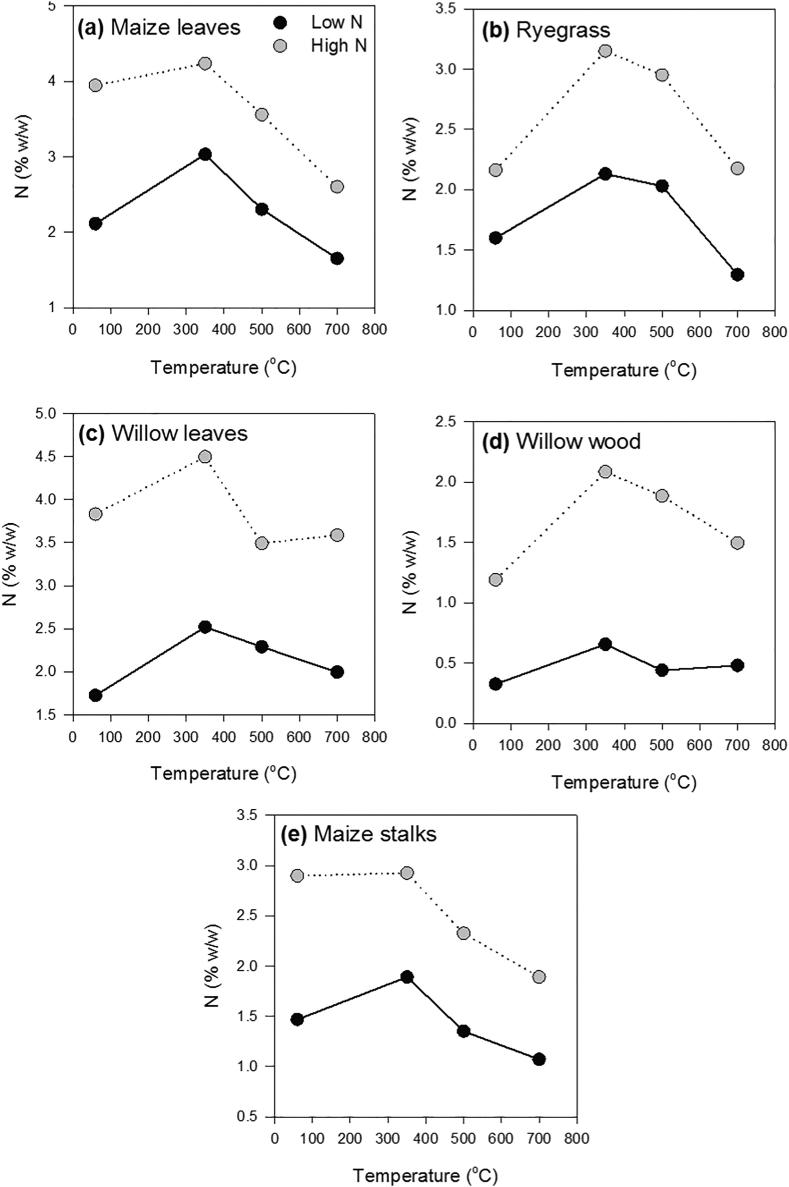
Nitrogen contents of uncharred OM and PyOM as a function of pyrolysis temperature. Nitrogen contents as a function of temperature for uncharred OM and PyOM of (a) maize leaves, (b) ryegrass, (c) willow leaves, (d) willow wood and (e) maize stalks, at three different pyrolysis temperatures. Grey circles and black circles represent high- and low-N content OM and PyOM, respectively.

Total N content in charred OM changed with both pyrolysis temperature and initial uncharred plant N contents (Fig. 1). At low OM-N, N contents of the uncharred OM increased by 29–98% when pyrolyzed to 350 °C; for high OM-N, the increase was only 1–43%. The PyOM-N increased up to the pyrolysis temperature of 350 °C, above which PyOM-N decreased as pyrolysis temperature progressed to 700 °C. The decrease in N content was similar for both low N-PyOM (26–77%) and high N-PyOM (25–63%) and was not affected by the type of original plant residue. However, the loss of N content was higher for high-N PyOM in absolute terms (Supplementary Fig. 4). Despite the N enrichment of PyOM with a pyrolysis temperature increase to 350 and 500 °C, its N content was not significantly different at 700 °C from that of the original OM-N (Supplementary Fig. 5).

The C:N ratios for uncharred OM varied between 24–138 and 11–38 for low-N OM and high-N OM, respectively (Supplementary Fig. 6). With increasing pyrolysis temperature to 350 °C the C:N ratio of low-N PyOM decreased for all types of OM (C:N of 22–108), suggesting an enrichment in N. As pyrolysis temperature increased to 700 °C, the C:N ratio for low-N PyOM increased to 36–168. On the other hand, the changes in C:N ratios for high-N PyOM were less noticeable. The C:N ratio decreased for willow wood due to an increase in N content, however for the remaining types of OM the C:N ratio slightly increased. This trend continued as pyrolysis temperature increased to 700 °C resulting in final C:N ratios that varied between 17–50. The lower variation in the C:N ratio for high-N PyOM suggested a lower N enrichment and subsequent loss with higher pyrolysis temperature when compared to low-N PyOM.

### 3.2 NEXAFS characterization of uncharred OM, PyOM, PyOM toluene extract and PyOM after toluene extraction

**Fig. 2:**
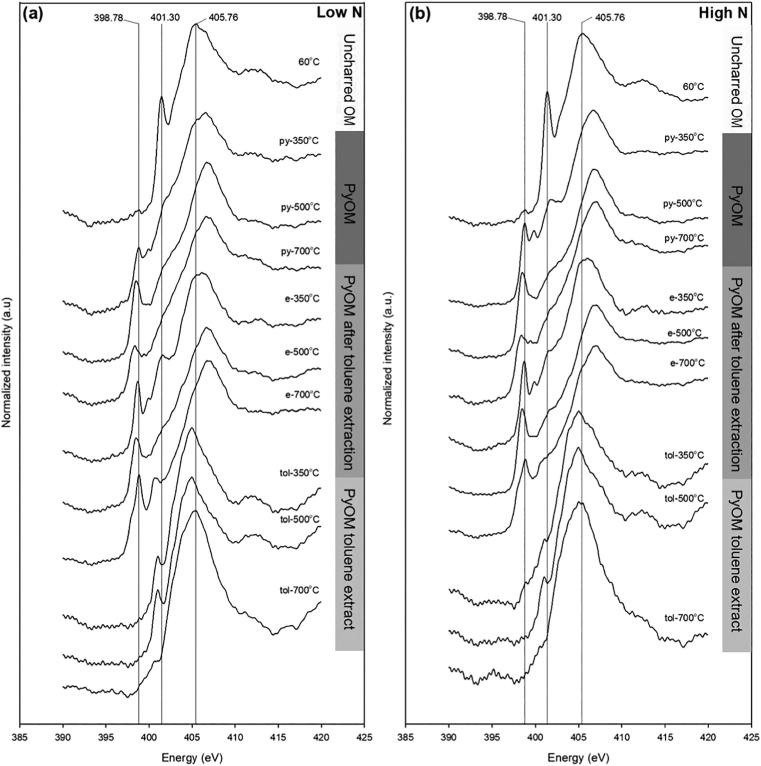
Nitrogen K-edge NEXAFS spectra of uncharred initial OM, entire PyOM (py-), extracted PyOM (e-), and the toluene extract of PyOM (tol-) as a function of pyrolysis temperature for maize leaves. (a) Low N-containing maize leaves, and (b) high N-containing maize leaves. Black lines represent the peak centers associated with selected key spectral features: 398.78 eV for C=N bonds in aromatic six-membered rings, 401.30 eV for amide N/C—N bonds in aromatic five-membered rings, and 405.76 eV for N—H bonds. Spectra of additional OM shown in Supplementary Figs. 7–10.

Nitrogen K-edge NEXAFS TEY spectra of uncharred OM, PyOM, PyOM toluene extract and PyOM after toluene extraction showed that the original uncharred OM-N content combined with thermal decomposition resulted in complex mixtures of N bonding environments for all the studied materials. Spectra of uncharred OM showed two distinct features irrespective of the type of plant and residue types (Fig. 2 and Supplementary Fig. 7–10). For uncharred maize leaves, a single narrow adsorption feature was present between 400.20 and 402.20 eV, with the peak center at 401.30 eV. Similar features were observed for the other uncharred plant residues (Supplementary Figs. 7–10). Deconvolution of the spectra indicates that the resolved feature corresponds to amide N or aromatic C—N of 5-membered rings (Supplementary Figs. 2–3), accounting for 54–64% of the 1s → π* region (Supplementary Tables 5–10). The NEXAFS spectra do not differentiate between amide N and aromatic C—N in 5-membered rings (also known as pyrrolic N in the literature) due to the high degree of resonance overlap (Leinweber et al., 2007). However, both N functional groups can be found in amino acids and proteins (Leinweber et al., 2007) which accounts for up to 87% of plant tissue (Hansson et al., 2003). The broader absorption band of uncharred OM near 405.76 eV represents N—H (Supplementary Figs. 2–3) bonds in the 1s → σ* transition from alkyl N (Leinweber et al., 2007, Gillespie et al., 2009) that are likely produced during charring rather than being a residue of the uncharred OM.

Total electron yield (TEY) spectra of PyOM, showed a loss of the amide N and aromatic C—N in 5-membered rings peak at 401.30 eV with an increase in pyrolysis temperature. At the same time, a region of aromatic C=N in 6-membered rings formed between 398.10–399.40 eV for all PyOM materials when compared to uncharred OM (Fig. 2 and Supplementary Figs. 7–10). The main 1s → π* peak for maize leaves pyrolyzed at 350 °C (PyOM-350 °C) was centered at 398.78 eV (Fig. 2a). As pyrolysis temperature increased to 500 °C, the aromatic C=N peak was resolved at 398.51 eV, whereas the same peak for PyOM heated to 700 °C was centered at 398.38 eV. The center of the main aromatic C=N peak for PyOM did not vary with OM residue type (Supplementary Figs. 7–10). However, the peak center generally shifted by 0.2 eV to lower energy levels with increasing pyrolysis temperature. One noticeable exception were the ryegrass spectra; the main aromatic C=N peak center did not change energy position between 500 °C and 700 °C. For PyOM-700 °C, there is a broadening of the aromatic C=N region causing the formation of a shoulder at approximately 397.90 eV. Spectral deconvolution revealed the aromatic C=N region included 6-membered rings (containing either one or two N atoms) and accounted for 9–35% of the 1s → π* region, depending on the type of OM (Supplementary Tables 6–10). Beyond the aromatic C=N region of 6-membered rings, other absorption bands were resolved at 399.93 eV for aromatic C=N of 5-membered rings. The spectra also showed a broad shoulder at 400.50–403.95 eV. The energy range of the shoulder suggests a mixture of bonding environments associated with quaternary aromatic-N, amide N in aromatic 6-membered rings, amide N/aromatic C—N from 5-membered rings (containing either one or two N atoms) and exocyclic N bonded to aromatic C (Supplementary Figs. 2–3).

The structure of PyOM after toluene extraction revealed four resolved peaks near 398.78, 399.93, 400.74, and 401.30 eV, corresponding to multiple 1s → π* bonding environments (Fig. 2 and Supplementary Figs. 7–10) These peaks represent bonding environments with aromatic C=N in 6-membered rings (containing either one or two N atoms), amide N in aromatic 6-membered rings and amide N/aromatic C—N in 5-membered rings (containing two N atoms). An additional peak was resolved at approximately 400.50 eV with increasing absorption intensity at higher pyrolysis temperature, representing a bonding environment known for quaternary aromatic-N. This peak was only resolved in the spectra for PyOM after extraction produced from maize stalks, willow leaves and wood.

For PyOM toluene extracts of all types of plant residues, the TEY spectra suggested that this fraction had a different bonding environment than both PyOM and PyOM after toluene extraction. Features with peak centers between 404.94–406.61 eV dominated the spectra (Fig. 2 and Supplementary Figs. 7–10). This spectral area is associated with alkyl N and 1s → σ* transition regions. It cannot be assigned to a specific N functional moiety due to high spectral overlap (Leinweber et al., 2007). A second but much smaller feature in the 1s → π* region is resolved in the spectra between 400.70–401.30 eV. This second region represents a bonding environment that includes amide N in aromatic 6-membered rings (containing two N atoms), amide N in non-aromatic C—N in 6-membered rings and amide N/aromatic C—N in 5-membered rings (containing two N atoms). Spectral deconvolution also showed a significant proportion of aliphatic C—NH_2_ bonded to aromatic 6-membered rings and C—NO_2_ bonded to aromatic 6-membered rings present in PyOM toluene extracts (Supplementary Tables 6–10), which are likely thermal condensation products rather than remnants of original plant OM (Debono and Villot, 2015). The average spectral deconvolution values for amide N in aromatic 6-membered rings (containing two N atoms) (7%) and exocyclic N bonded to aromatic 6-membered rings (28%) were significantly different (LSMeans contrast, *P* < 0.0001) for the PyOM toluene extract when compared to PyOM and PyOM after toluene extraction (Supplementary Tables 6–10).

### 3.3 Temperature and N content effects on PyOM-N

**Fig. 3:**
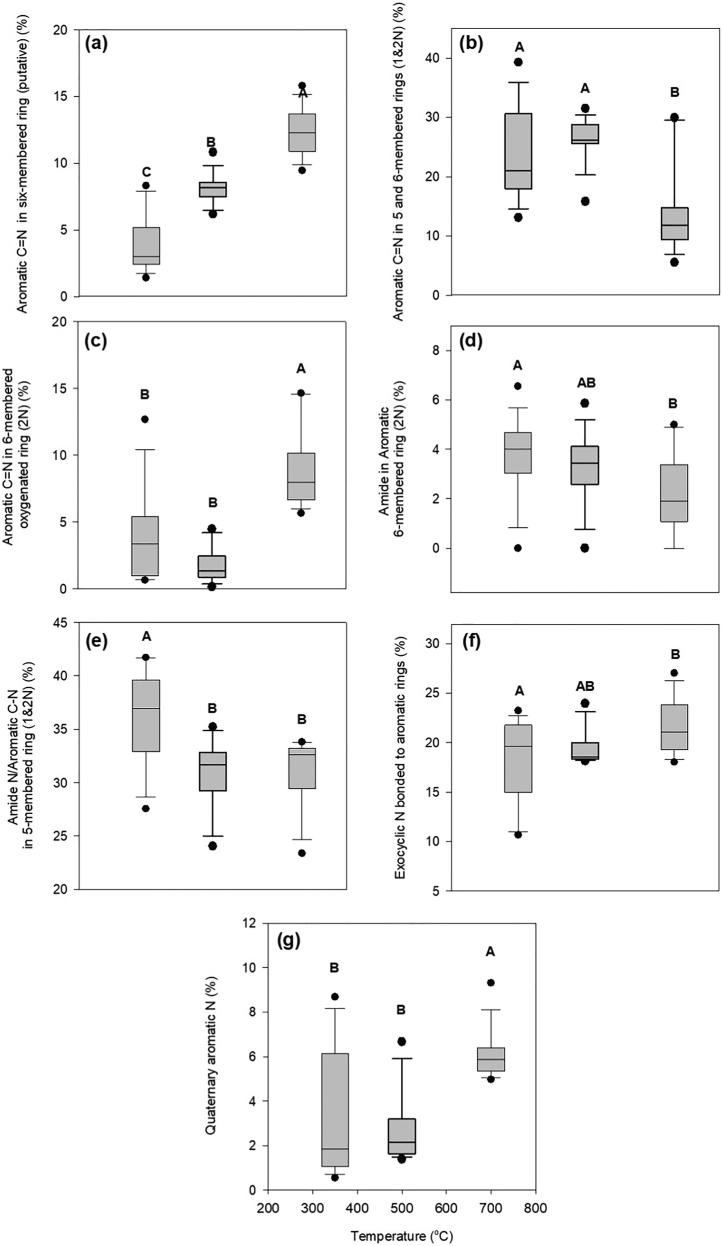
Proportion of aromatic nitrogen functional groups for PyOM at different pyrolysis temperatures. Proportions of aromatic nitrogen functional groups aggregated across all PyOM prepared from different OM residues. (a) Aromatic C=N in 6-membered rings represents the proportion of the total spectral absorption intensity corresponding to spectral features at 398.10 eV, (b) aromatic C=N in 5 and 6-membered rings containing 1 and 2 N atoms, represents the proportion of the total absorption intensity of the sum of spectral features at 398.78 and 399.93 eV, (c) aromatic C=N in 6-membered oxygenated rings represents the proportion of the total absorption intensity corresponding to spectral features at 399.40 eV, (d) amide N in an aromatic C—N 6-membered ring represents the proportion of the total absorption intensity spectral feature at 400.74 eV, (e) amide N/aromatic C—N in rings with delocalized pairs of electrons from aromatic N from 5-membered rings (containing one or two N atoms) represents the proportion of the total absorption intensity of the sum of spectral features at 401.30 and 402.13 eV, (f) exocyclic N bonded to aromatic 6-membered rings represents the proportion of the total absorption intensity of the sum of spectral features at 403.00 and 403.61 eV, and (g) quaternary aromatic N represents the proportion of the total absorption intensity spectral feature at 400.9 eV.

Pyrolysis temperature had a significant effect (LSMeans contrast, *P* < 0.0001) on the proportion of different N functional groups present in PyOM (Fig. 3) for all types of OM residues. As pyrolysis temperature increased from 350 °C to 700 °C, aromatic C=N in 6-membered rings (putative) (398.10 eV) increased threefold. Aromatic C=N in 6-membered oxygenated rings increased sevenfold, whereas quaternary aromatic N (400.90 eV) doubled. In contrast, aromatic C=N in 5- and 6-membered rings (containing one and two N atoms), amide N in aromatic 6-membered rings, and amide N/aromatic C—N in 5-membered rings (containing either one or two N atoms) decreased with increasing pyrolysis temperature by 50, 42 and 15%, respectively. Exocyclic N bonded to aromatic 6-membered rings increased by 18% with increasing pyrolysis temperature.

**Fig. 4:**
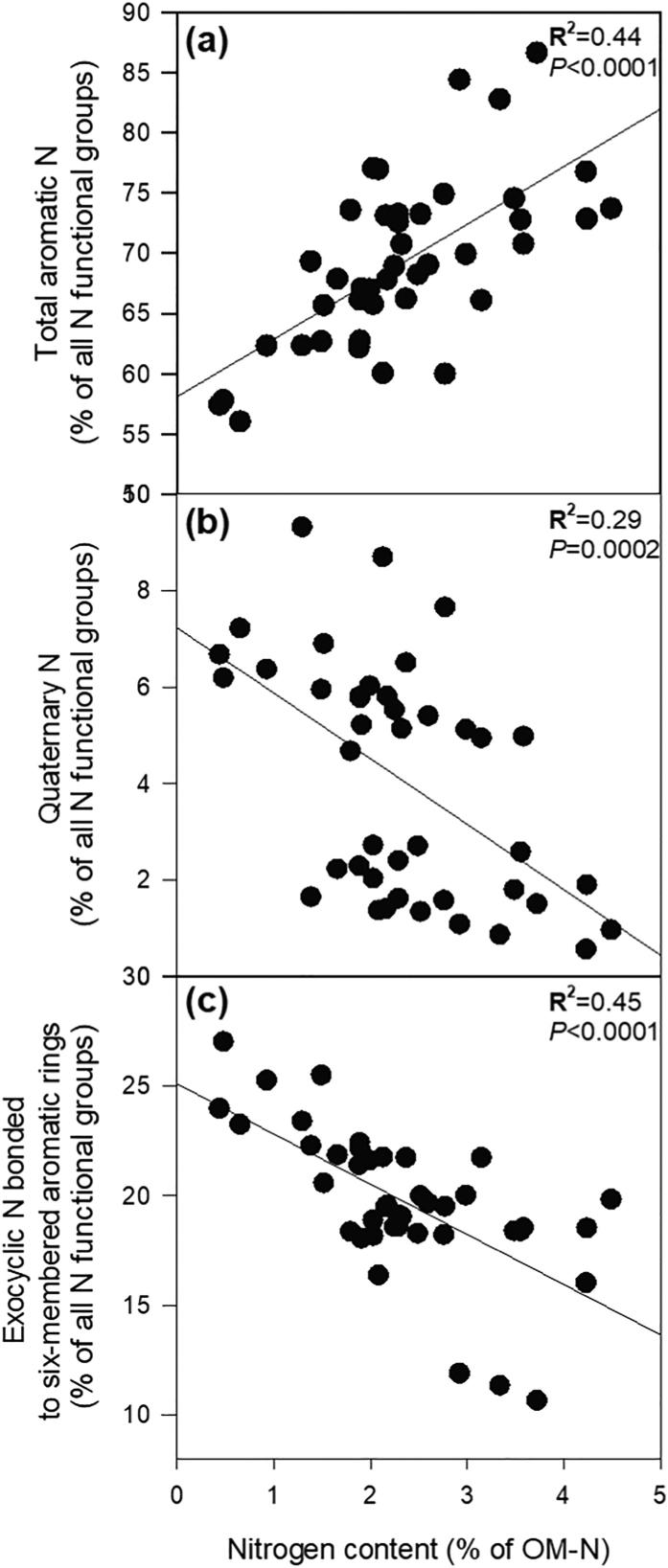
Effects of OM nitrogen contents on the formation of N functional groups in PyOM. (a) Sum of all aromatic N heterocycles as a function of the total of all N functional groups in PyOM, (b) proportion of quaternary aromatic N as a function of the total of all N functional groups in PyOM, and (c) sum of all N exocyclic N attached to a 6-membered aromatic ring as a function of the total of all N functional groups in PyOM expressed as a proportion of the total absorption intensity (left y-axis), against the amount of N in the original OM. Plots are fitted with significant regression lines: $\mathrm{Proportion}\;of\;total\;aromatic\;N\;in\;PyOM-N=58.1+4.78\;OM-N,\;R^{2}=0.44,P<0.0001$; $\mathrm{Proportion}\;of\;quaternary\;N\;in\;PyOM-N=7.2-1.36\;OM-N,\;R^{2}=0.29,\;P=0.0002$; $\mathrm{Proportion}\;of\;exocyclic\;N\;bonded\;to\;6-membered\;aromatic\;rings\;in\;PyOM-N=25.1-2.29\;OM-N,\;R^{2}=0.43,\;P<0.0001.$

The initial OM-N contents of all types of residues had a significant and positive relationship with the proportion of total aromatic N heterocycles (sum of all aromatic heterocycles including 5 and 6-membered rings) in PyOM (Fig. 4a). On the other hand, N contents negatively correlated with quaternary aromatic N (Fig. 4b) and exocyclic N bonded to aromatic 6-membered rings (Fig. 4c) in PyOM.

### 3.4 Carbon and N mineralization of OM and PyOM

**Fig. 5:**
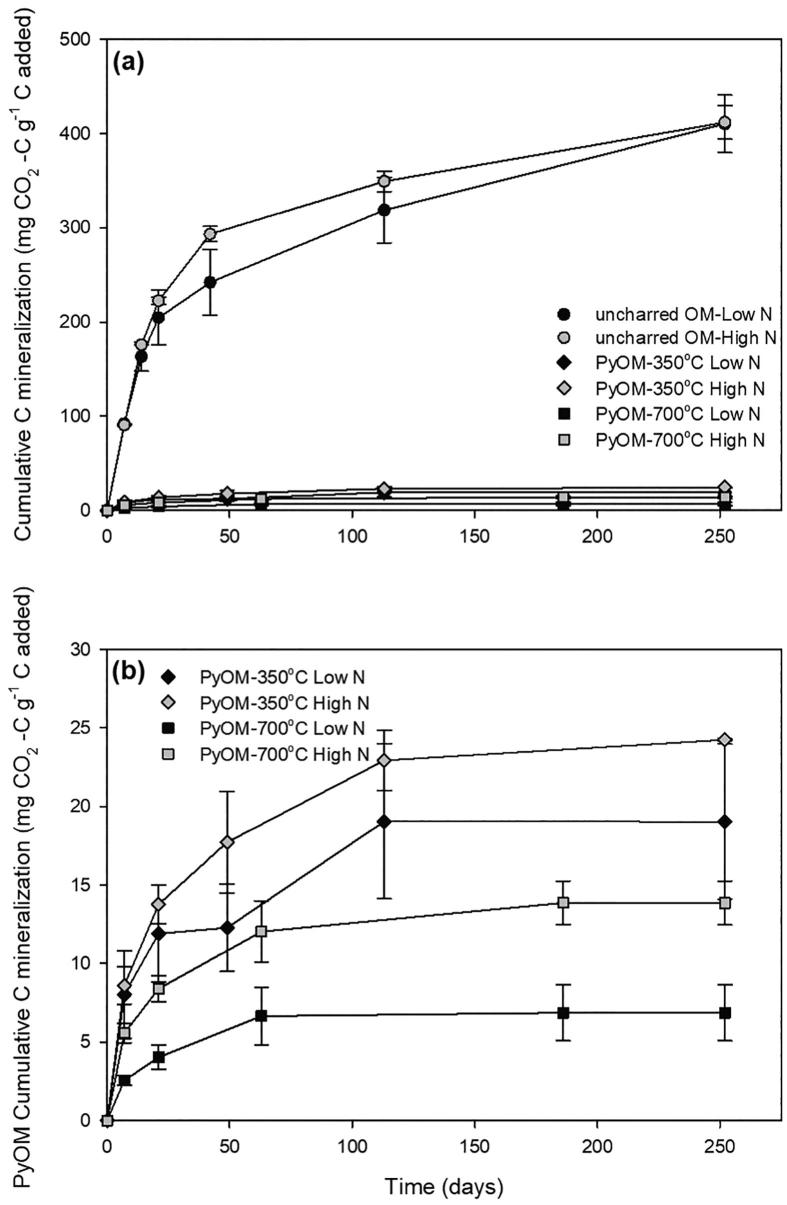
Cumulative C mineralized as a proportion of added C. (a) Cumulative C mineralization over time for maize leaves dried at 60 °C and PyOM pyrolyzed at 350 °C and 700 °C, and (b) cumulative C mineralization for PyOM pyrolyzed at 350 °C and 700 °C. Black circles, diamonds and squares represent high N-containing maize leaves whereas grey circles, diamonds and squares represent low N-containing maize leaves.

**Fig. 6:**
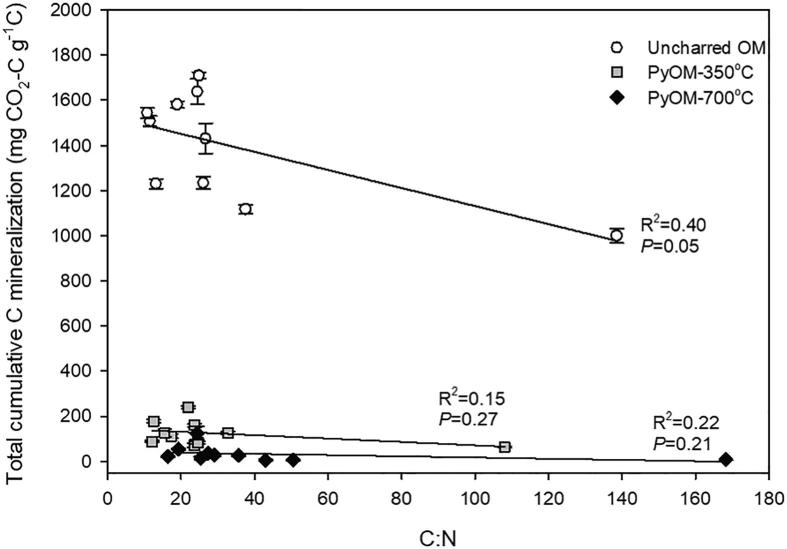
Relationship between the carbon-to-nitrogen (w/w) ratio and the mineralization of OM-C or PyOM-C, respectively. White circles, grey squares and black squares represent OM, PyOM at 350 °C and 700 °C, respectively. Lines indicate linear regression: OM-C = $1.53-0.004x,P=0.05,R^{2}=0.40$ ; PyOM-C at 350 °C: $y=0.15-0.0008x,P=0.27,R^{2}=0.15$; PyOM-C at 700 °C: $y=0.03-0.0002x,P=0.21,R^{2}=0.22$.

Cumulative mineralization of OM-C for all types of residues was higher than that of PyOM-C (Fig. 5a and Supplementary Figs. 11a–14a). Cumulative PyOM-C mineralization throughout the 252 days of incubation was approximately two times greater for PyOM-350 °C than for PyOM-700 °C (Fig. 5b and Supplementary Figs. 11b–14b). Total cumulative C mineralization was highest for OM-C (999–1709 mg CO_2_-C g^−1^ C) and lowest for PyOM-700 °C (5–125 mg CO_2_-C g^−1^ C). Total cumulative C mineralization for uncharred OM-C was 10–43 times greater (LSMeans contrast, *P* < 0.0001) compared to PyOM-C for all PyOM. Total mineralization of PyOM-C decreased by 75% with an increase in pyrolysis temperatures from 350 °C to 700 °C (*P =* 0.0003). The C:N ratios of uncharred OM were significantly (*P =* 0.05) and negatively correlated to the uncharred OM-C mineralization (Fig. 6), albeit relying to a great extent on a single data point. In contrast, PyOM-C mineralization was not correlated to the C:N ratio for both pyrolysis temperatures even with one type of PyOM having high C:N.

Mineral N contents were 13–78 times higher for all uncharred OM when compared to PyOM. For most of the types of OM residues, net N mineralization (i.e., increase in mineral N by microbial activity) occurred during the incubation. However, all PyOM at all pyrolysis temperatures showed net N immobilization (i.e., N uptake by soil microorganisms) during the incubation (Supplementary Fig. 15).

### 3.5 PyOM-C mineralization and PyOM-N correlation

**Fig. 7:**
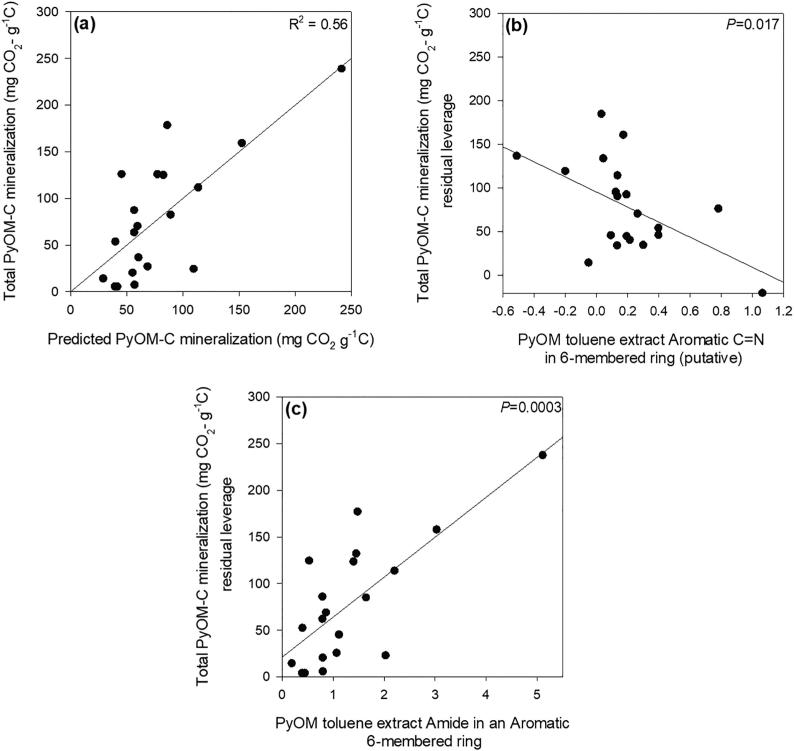
Relationship between cumulative PyOM-C mineralized and ratios of N functional groups from the PyOM toluene extract as observed by N K-edge NEXAFS. (a) Actual vs. multiple regression model predicted response values for cumulative PyOM-C mineralized; Total PyOM-C mineralized: y=38.18-86.12∗AromaticC=Nin6memberedrings+42.80∗AmideNinaromatic6memberedrings. (b) relationship between cumulative PyOM-C mineralized and aromatic C=N in 6-membered rings, and (c) amide N in aromatic 6-membered rings. Removing one outlying data point decreased the R^2^ for (a) to 0.33 (*P* = 0.04) and the *P* value of (b) and (c) to 0.06 and 0.01, respectively.

The abundance of C=N 6-membered rings present in PyOM had a negative and strong correlation with PyOM-C mineralization for all pyrolysis temperatures (Supplementary Fig. 16). The N functionalities in PyOM after toluene extraction (Supplementary Fig. 17) and PyOM toluene extract (Fig. 7) were also correlated to PyOM-C mineralization. Results obtained by multivariate data analysis strongly suggest that aromatic C=N in 6-membered rings (putative) and amide N in aromatic 6-membered rings found in PyOM toluene extracts were highly correlated to the total cumulative mineralization of PyOM-C (Fig. 7). PyOM-C mineralization was positively correlated to the presence of amide N in aromatic 6-membered rings, which explained 37% of the variance in the model. The sequential inclusion of aromatic C=N in 6-membered rings (putative) accounted for the remaining 18% of the variance in the model and was negatively correlated with PyOM-C mineralization.

For the remaining PyOM after toluene extraction, C=N in 6-membered rings (putative) was significantly (*P =* 0.0006) and negatively correlated to PyOM-C mineralization (Supplementary Fig. 17). C=N in 6-membered rings alone accounted for 49% of the variance in the model.

## 4 Discussion

### 4.1 Pyrolysis temperature transforms the molecular structure of PyOM-N

Ample evidence supports the effects of charring temperature on the final molecular structure and N content of PyOM (Knicker et al., 1996, Almendros et al., 2003, Chen et al., 2014). NEXAFS spectra in our study showed the transformation of OM-N from amide N/5-membered aromatic N heterocycles (likely from amino acids and peptides) to aromatic 6-membered N heterocycles and quaternary aromatic N. Our findings agree with various studies (Knicker et al., 1996, Almendros et al., 2003, Knicker, 2010, Chen et al., 2014) that report a high degree of aromatic N in PyOM. The initial transformation of OM-N to amide N/5-membered aromatic N heterocycles is consistent with Chen et al., 2014, Chen et al., 2017 who reported that the thermal conversion of protein N can result in the formation of 5-membered aromatic N heterocycles (Sharma et al., 2004, Sharma et al., 2009, Ren et al., 2011, Ren and Zhao, 2012). Higher pyrolysis temperatures result in an increase in the aromatic 6-membered N heterocycles (pyridines) and concurrent decrease of five-membered aromatic N heterocycles (pyrroles). This would suggest that pyrroles are transformed to pyridines at higher pyrolysis temperatures via ring expansion (Patterson and Drenchko, 1962, Pels et al., 1995). Lifshitz et al. (2003) demonstrated the transformation of methylpyrroles to pyridines via ring expansion. Free radical reactions produce unstable intermediates that can experience ring expansion by accepting the insertion of methylene groups at C—C or C—N single bonds to produce pyridines (Dubnikova and Lifshitz, 2000, Lifshitz et al., 2003).

Quaternary aromatic N as observed in our study is not commonly reported as a constituent of PyOM-N but mostly found in coal (Pels et al., 1995, Schmiers et al., 1999, Xiao et al., 2005). However, recent studies (Chen et al., 2017) have reported the formation of quaternary aromatic N during the pyrolysis of algae between 400 and 800 °C. Quaternary aromatic N (also known as graphitic N or substitutional N in the literature) is the result of substituting an aromatic C for a N atom, which is bonded to three adjacent C within the polyaromatic structure (Pels et al., 1995, Zhao et al., 2015, Chen et al., 2017). The final molecular configuration results in excess valence charge of the quaternary N atom which becomes delocalized leading to a lower effective charge at the N atom (Zhang et al., 2016, Manojkumar et al., 2019). Our data confirmed this possible configuration since the N K-edge NEXAFS spectra show that aromatic N heterocycles transform with increasing pyrolysis temperature (Fig. 3). Quaternary N is likely synthesized from the transformation of C=N in 6-membered rings even at low temperatures. As pyrolysis temperature increases quaternary N produced at low pyrolysis temperatures is preserved as temperatures increase due to its thermal stability (Xiao et al., 2005).

### 4.2 Uncharred organic matter nitrogen content influences molecular structure of PyOM-N

Thermal conversion of OM-N was responsible for the high aromaticity of N and enrichment of N in PyOM, in our study, however, temperature alone did not account for differences in the formation of aromatic N heterocycles. Our results suggest that the initial OM-N content was correlated with the proportion of aromatic N heterocycles present in PyOM-N (Fig. 4a). Studies on N-doped C allotropes (Bhattacharyya et al., 2002, Chen et al., 2010, Jian et al., 2013, Bulusheva et al., 2015) reported a greater abundance of aromatic N heterocycles when allotropes were exposed to higher amounts of exogenous N. However, the incorporation of exogenous N to previously formed C allotropes may proceed through potentially different reaction pathways than those of endogenous N already present in OM as in our study. To our knowledge, there is no prior confirmation on how the initial N content in OM is correlated with the proportion of aromatic heterocyclic N structures as a fraction of PyOM-N during pyrolysis.

Several mechanisms may be responsible for an increase in the proportion of aromatic N in PyOM-N with increasing OM-N contents (Fig. 4a). Pyrolysis of amino acids demonstrates the complex chemistry behind the formation of pyrolysis products (Sharma et al., 2003, Sharma et al., 2004). Amino acids with reactive polar side chains can form 5-, 6- or 7-membered aromatic N heterocyclic compounds (Chiavari and Galletti, 1992, Sharma et al., 2004, Choi and Ko, 2011). However, the formation of aromatic N heterocycles in PyOM is much more complex than that of pure amino acid pyrolysis.

An alternative theory to the formation of aromatic N heterocycles in PyOM follows the chemistry of Maillard reactions, where sugars and amino acids react during pyrolysis to form N-substituted polyaromatic rings (Darvell et al., 2012). Organic matter is a mixture of mostly proteinaceous and aliphatic N along with cellulose, hemicellulose, and lignin (Ren et al., 2011, Ren and Zhao, 2012), which react during pyrolysis to produce aromatic N heterocycles. Multiple studies on the pyrolysis of mixtures of sugars and amino acid model compounds (Britt et al., 2004, Moens et al., 2004, Sharma et al., 2009, Darvell et al., 2012) have reported the formation of N-substituted polyaromatic rings and explained these with Maillard reactions. It has been shown that the decomposition of the intermediate Amadori compound during pyrolysis was responsible for aromatic N heterocycles, mostly found as aromatic C=N in 5-membered rings (pyrroles in the literature) (Shigematsu et al., 1977, Britt et al., 2004, Moens et al., 2004, Sharma et al., 2009) and to a lesser degree as aromatic C=N in 6-membered rings. These findings are primarily valid for gas-phase N transformations and might not explain solid-state reactions. In our study, we examined the molecular structure of the solid-state products during pyrolysis. If Maillard reactions were a primary pathway for the formation of N heterocycles in PyOM, we would have expected to see a resolved peak at approximately 401.30 ± 0.12 eV. This peak represents the aromatic C=N 5-membered heterocycles that are a function of the degradation of Amadori compounds during pyrolysis (Yaylayan, 1990, Yaylayan et al., 1994). However, our PyOM spectra (Fig. 2) showed a broad unresolved shoulder at 401.30 eV, which suggests that the main Amadori decomposition products were not present in PyOM. Hence, Maillard reactions may not be the dominant pathway for the formation of aromatic N heterocycles in our study. In contrast, PyOM toluene extract spectra indeed had a well-resolved peak in this region suggesting that the re-condensed pyrolysis gases in our study could be a product of gas-phase Maillard reactions, similar to those reported in previous studies cited above. To confirm this explanation, further solid-state and especially gas-phase pyrolysis studies of OM-N are needed.

Several other studies (Jian et al., 2013, Bulusheva et al., 2015, Chen et al., 2017) have concluded that the molecular structure of the N precursor plays a significant role in the formation of aromatic N heterocycles. These studies implied that if the precursor N is present in an aromatic ring, the final pyrolysis product will contain aromatic N heterocycles. In the present study, the bulk of OM-N was consistent with amide N/C—N in 5-membered rings from the presence of peptides bonds and amino acids with heterocyclic structures. However, no correlation exists between the proportion of aromatic N heterocycles in OM-N and PyOM-N in our study. Although our data did not show any correlation between the precursor N structure and the final PyOM-N structure, this does not conclusively exclude this reaction pathway. It may not be the dominant pathway to the increased proportion of aromatic N heterocycles in PyOM.

We propose an alternate explanation for the positive correlation between OM-N and aromatic N heterocycle contents in PyOM-N based on the energetics of the incorporation of N to highly condensed aromatic C. For our study, as N contents in the original OM increased, N atoms during charring were preferentially incorporated into aromatic sites (Fig. 4a). This phenomenon could be associated with the energy needed to add N atoms into the PyOM-C network. The molecular structure of PyOM is heterogeneous, containing areas that include both amorphous and graphitic C (Keiluweit et al., 2010). As the degree of condensation increases in PyOM, the incorporation of N into the C network becomes more energy intensive due to the molecular and geometric structure of PyOM-C. The energy barrier associated with incorporating N atoms is high due to the atomic coordination of graphitic C domains. The atomic coordination confers a planar geometry to graphitic C which is hardly disrupted at high C:N. However, as N increases in PyOM, the atomic configuration of PyOM-C can be disrupted and the energy barrier associated with further N incorporation is lowered (dos Santos and Alvarez, 1998, Chen et al., 2010), allowing for more N to be incorporated. These studies have been conducted in C allotropes exposed to higher exogenous N which are not necessarily found in OM. However, this theoretical principle of N incorporation presented by dos Santos and Alvarez (1998), might be possible due to the molecular heterogeneity found in PyOM, where both amorphous and graphitic C can be found. Further studies on the electronic and atomic configuration of PyOM-C and N are necessary to elucidate how increasing N content can facilitate N incorporation into the C network.

### 4.3 Nitrogen form effects on PyOM-N and PyOM-C mineralization

The molecular and structural change of OM-N during thermal degradation affected the availability of N and subsequent microbial oxidation of PyOM. Uncharred OM exhibited net N mineralization (Supplementary Fig. 15a), whereas PyOM experienced net immobilization of N for PyOM at 350 °C and 700 °C (Supplementary Fig. 15b–c), despite the fact that the N content of the bulk PyOM did on average not decline compared to that of the original uncharred OM. The change from N mineralization to immobilization with increasing pyrolysis temperature can be best explained by the molecular and structural change experienced by N during thermal degradation, instead of by C:N stoichiometry or total N content. PyOM-N exhibits a highly aromatic structure, reducing N availability for microbial degradation. PyOM-N immobilization trends have been reported by others (Novak et al., 2010, Wang et al., 2012b, Chen et al., 2014); however, the effect is considered short-lived (Bruun et al., 2012) and mineralization of aromatic N heterocycles in PyOM is possible on longer time scales (Hilscher and Knicker, 2011, de la Rosa and Knicker, 2011). Our N mineralization study lasted 50 days and therefore, the net N immobilization observed has to be seen in this context and a switch from immobilization to mineralization may be observed over longer periods of time.

In contrast, we observed PyOM-C mineralization during our study, which indicated that a fraction of PyOM-C was accessible to microbes. Several studies (Keiluweit et al., 2010, Deenik et al., 2010, Smith et al., 2010) have identified re-condensed volatiles found on the surface and in the pores of PyOM to contain easily mineralizable PyOM-C (Smith et al., 2010) and PyOM-N (Deenik et al., 2010, Wang et al., 2012a). Our multivariate regression model suggests that PyOM-C mineralization in our study is mostly controlled by aromatic N in 6-membered heterocycles as well as amide N in aromatic 6-membered rings found in the PyOM toluene extract. While aromatic N in 6-membered heterocycles in PyOM extracts reduced the mineralization of PyOM-C, amide N in aromatic 6-membered rings stimulated PyOM-C mineralization.

We propose that aromatic N heterocycles in PyOM controlled PyOM-C mineralization via the location of N atoms. First, the molecular structure of the fraction of PyOM where N heterocycles are present had an effect on PyOM-C mineralization. The aromatic nature of N in PyOM extracts may at first glance suggest that this type of N is not available for microbial use. However, microbial metabolization of aromatic N heterocycles is indeed possible via hydroxylation of the aromatic ring catalyzed by mono- or dehydrogenase in the presence of oxygen, followed by ring opening (Fetzner, 2000). The microbial degradation of aromatic N heterocycles can yield aliphatic N compounds (Sims et al., 1989, Fetzner, 2000) readily available for microbial use. The transformation of both C and N through this process in turn allows the release of CO_2_-C.

Secondly, the location of N atoms within the C network can also regulate the mineralization of PyOM-C. This is supported by the apparent contradiction that aromatic C=N in 6-membered rings reduced PyOM-C mineralization, yet amide N in aromatic 6-membered rings stimulated C mineralization. The different role of these two moieties in the control of PyOM-C mineralization can be best explained by the position of the N atom within the C structure of PyOM. Aromatic C=N in 6-membered rings can be found in the C backbone of PyOM (PyOM after extraction in our study) and was in fact the principal moiety controlling the mineralization of PyOM-C (Supplementary Fig. 17). Nitrogen atoms in this PyOM fraction are likely substitutes in the structure of amorphous C (Keiluweit et al., 2010, Knicker, 2010). Therefore, N atoms may be physically protected by highly condensed C domains and less likely to be oxidized. The amide N in aromatic 6-membered rings was present in the PyOM toluene extract. Such aromatic N groups bearing an amide group are most likely found in the surface structure of PyOM. Several studies (Arrigo et al., 2010, Wang et al., 2012a) have shown that oxygenated functional groups are preferentially located at the edge of the C network and may therefore be more easily metabolized by microorganisms.

## 5 Conclusion

Differences in plant N contents were shown to have significant effects on the geochemistry of fire-derived OM. An enrichment in N during fires has important consequences for the withdrawal of N from the reactive global N cycles. Geochemically, the observed enrichment of N in PyOM is best explained by the incorporation of N atoms into heterocyclic aromatic moieties during fire rather than remnants of aromatic N structures in plant residues. While charring temperature has previously been considered as the dominant control in the formation of aromatic N heterocycles, our study demonstrates that the initial N content of the organic matter is equally important in the formation of these moeities. In addition, the structural transformation of N in PyOM contributes to the persistence of PyOM in the environment through stabilization of PyOM-C. Carbon mineralization of PyOM in the present study is affected by the molecular structure of N in different fractions of PyOM. Oxidized forms of N heterocycles found in the chemically extractable fraction of PyOM may explain the short-term increase in PyOM-C mineralization seen in most studies. In contrast, aromatic N heterocycles embedded in the C network of PyOM lead to lower mineralization of PyOM-C. These findings support the hypothesis that the geochemical forms of N in fire-derived PyOM rather than total N concentrations or C:N ratios play a major role in the persistence of PyOM in the global C cycles. The results may also guide engineering biochars with application in environmental remediation, nutrient recycling and sequestration of atmospheric CO_2_. Future research is needed to combine decomposition experiments and analytical tools for molecular characterization in situ for a wide variety of char residues and their changes over the short and long term.

## Declaration of Competing Interest

The authors declare that they have no known competing financial interests or personal relationships that could have appeared to influence the work reported in this paper.
