# Supplementary material for: Nitrogen speciation and transformations in fire-derived organic matter
Source: Geochim Cosmochim Acta. 2020 May 1;276:170–85. doi: 10.1016/j.gca.2020.02.034 (PMC7171705; doi:10.1016/j.gca.2020.02.034)
Supplement: Supplementary data 1 [file mmc1.docx]

**Supplementary Material**

**Nitrogen speciation and transformations in fire-derived organic matter**

Dorisel Torres-Rojas^a^, Rachel Hestrin^a^, Dawit Solomon^a,d^, Adam W. Gillespie^b^, James J. Dynes^c^, Tom Z. Regier^c^, Johannes Lehmann^a,e,*^

^a^Soil and Crop Sciences, Cornell University, Ithaca, NY 14853, USA

^b^School of Environmental Sciences, University of Guelph, Guelph ON, Canada

^c^Canadian Light Source Inc., Saskatoon, SK, Canada

^d^CGIAR Research Program on Climate Change, Agriculture and Food Security (CCAFS), P.O. Box 5689. Addis Ababa, Ethiopia

^e^Atkinson Center for a Sustainable Future, Cornell University, Ithaca, NY 14853, USA

**corresponding author:* phone: 607-254-1236; fax: 1-607-255-3207 email: [*CL273@cornell.edu*](mailto:CL273@cornell.edu)

**1. Supplementary Materials and Methods**

**1.1. Plant residues**

Maize- *Zea mays* L., ryegrass- *Lollium perenne* L., and willow- *Salix viminalix* L. plants were grown under controlled conditions in a greenhouse. A mixture of peat moss, vermiculite and perlite (1:1:1 per volume) was used as growing media. Each plant species was fertilized with a customized Hoagland’s solution with varying concentrations of N. Eight different N concentrations were used each adjusted to the specific plant species.

Due to different rates of dry matter accumulation between plant species, aboveground biomass was harvested on different dates. Ryegrass was grown for approximately 30 days, and leaf blade clippings were harvested twice during the entire growth period. Maize was grown for 28 days and willow was allowed to grow and pruned every 30 days to allow for new growth.

**1.2. NEXAFS sample preparation**

Solid samples (i.e., PyOM and PyOM after extraction) were mixed with deionized water (0.5 mg mL^-1^ water) in 1.5-mL Eppendorf vials to obtain a slurry. Samples were thoroughly mixed using a vortex mixer to achieve homogenous wetting. Using a pipette, an aliquot of 4 μL was deposited onto Au coated Si wafers and air-dried at room temperature. Toluene extract solution was directly deposited and dispersed onto an Au-coated Si wafer. The toluene was allowed to evaporate under atmospheric conditions under a hood resulting in a thin layer of extract. Wafers were affixed to the sample plate using double-sided carbon tape before insertion into the absorption chamber.

**1.3. NEXAS spectral deconvolution**

The spectral deconvolution method was designed to provide a platform where N K-edge spectral differences could be evaluated across a wide-ranging set of organic materials with varying molecular composition. An initial model was built consisting of 30 Gaussian functions with fixed energies which were derived from reference compounds measured in this study (Supplementary Tables 3-4). To reduce the degrees of freedom for the fit, the half-width at half-maximum (HWHM) was fixed at 0.4 eV for all Gaussian functions in the 1s → π^*^ region. Past the edge step, in the 1s → σ^*^ transition, spectral features become broad due to delocalized excited states (Le Guillou et al., 2018). Therefore, HWHM values were fixed at 2.00 eV and the height of the Gaussian functions were allowed to float. Experimental spectral data were modeled using the Levenberg-Marquardt non-linear least-squares fitting method between 390 and 420 eV. After initial fit, Gaussian functions with an area less than 0.1 were eliminated from the model. The reduced model was iterated until the remaining Gaussian functions had an area greater than 0.1. The modeling exercise resulted in a reduced model for each experimental spectra.

A second modeling process was executed on each experimental spectrum with the reduced model from the previous fitting process. The height of the Gaussian function was unconstrained and the HWHM was constrained between 0.4±0.2 eV to achieve an optimal fit. The experimental data were then fitted using the same non-linear least-squares fitting method as before and iterated until all remaining Gaussian functions had an area greater than 0.1. The resulting model for each experimental spectrum was then used to compare between contrasting organic materials.

Due to high overlap in the 1s → σ^*^ transition, spectral features in this region were modeled but not used in the final analysis. Only the spectral features in the 1s → π^*^ region were used for analysis. For comparison between varying organic materials, the proportion of the total area of the 1s → π^*^ region for each Gaussian function was determined.


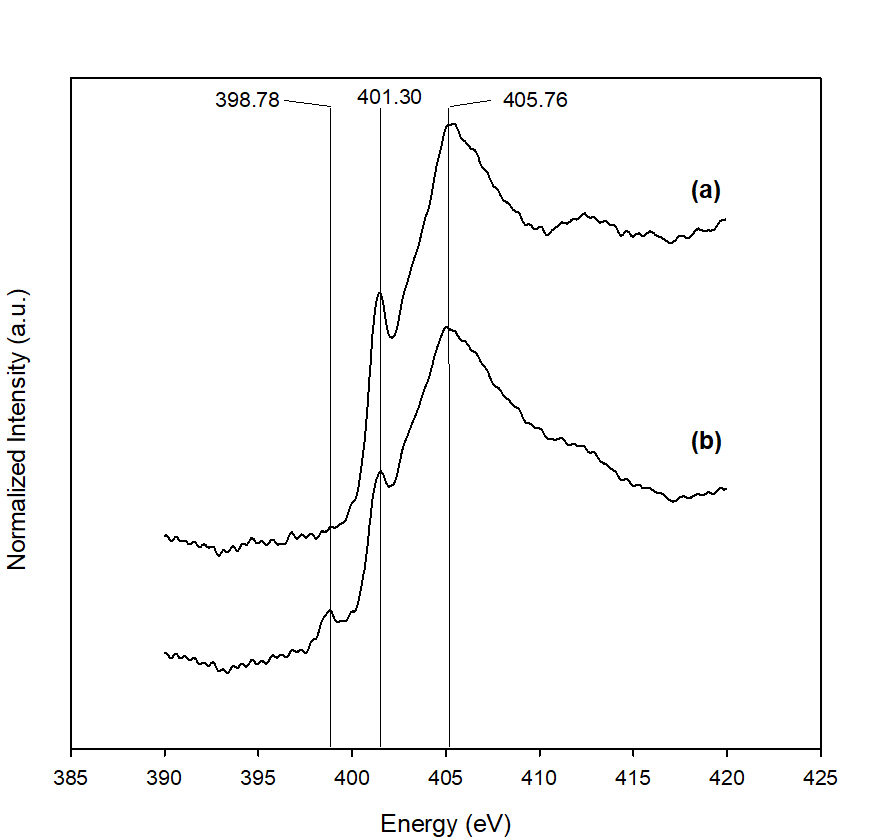


**Supplementary Figure 1.** Test for radiation damage by N K-edge TEY NEXAFS spectroscopy of uncharred OM. (a) Averaged spectra for uncharred OM, scanned over 5 different locations in the sample as performed for the remainder of the NEXAFS analyses; and (b) spectra of uncharred OM scanned on the same location multiple times (n=12), developed a new spectral feature at 398.78 eV.

**
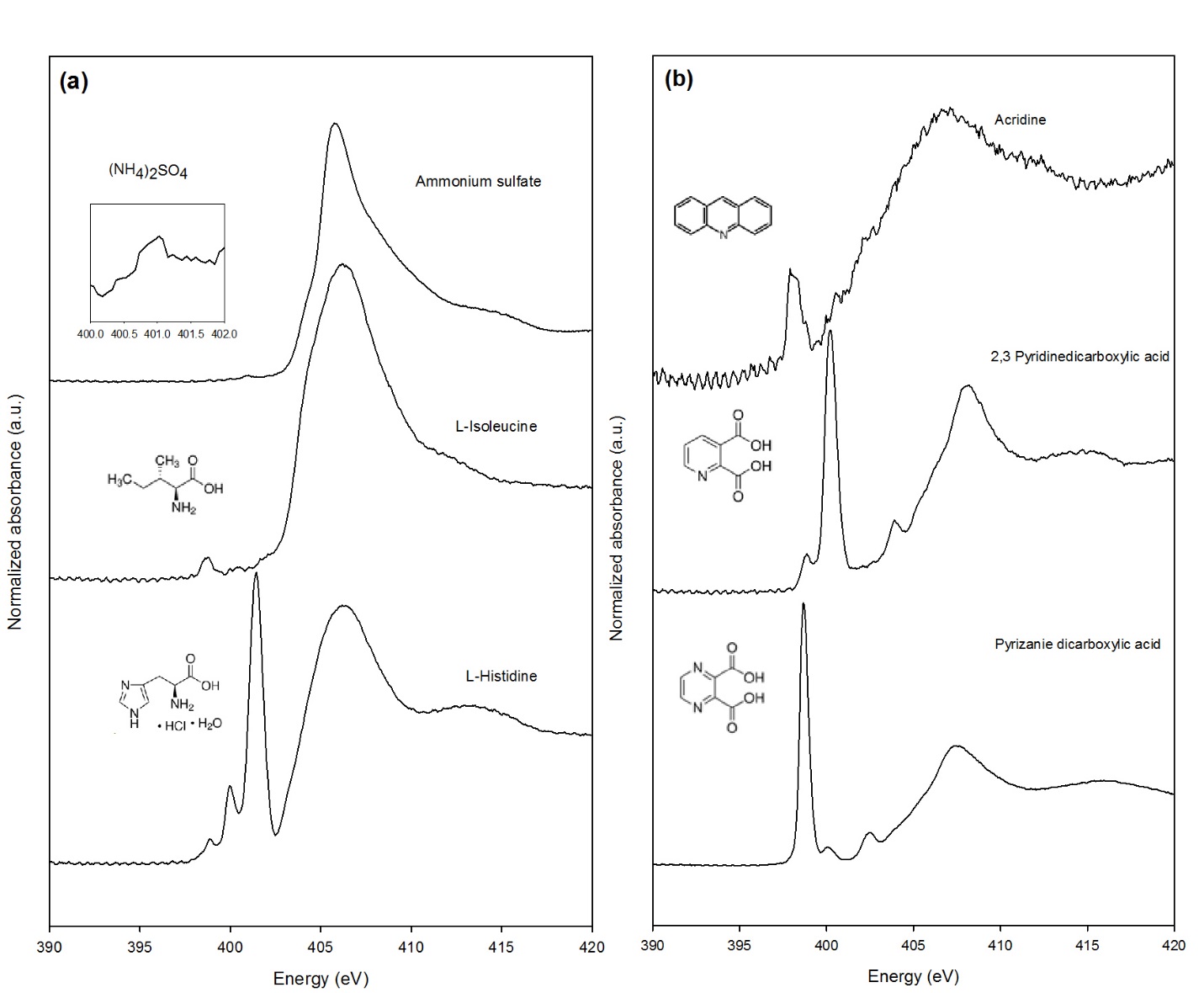
**

**Supplementary Figure 2.** Nitrogen K-edge TEY NEXAFS spectra of N standard compounds. (a) Mineral N and amino acid compounds. (b) 6-membered heterocycles with one or two N substitutions.

**
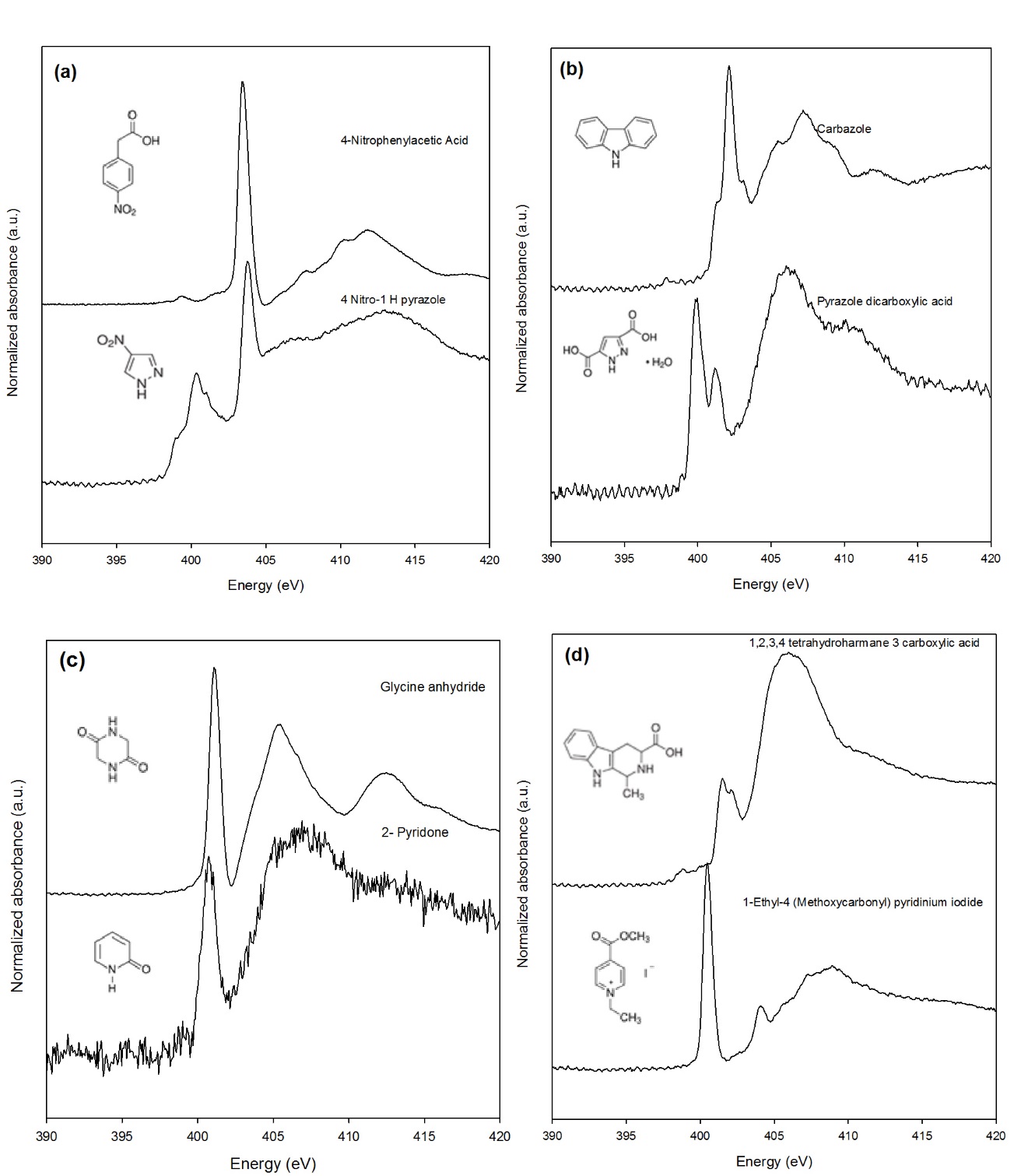
**

**Supplementary Figure 3.** Nitrogen K-edge TEY NEXAFS spectra of N standard compounds. (a) Nitro functional groups. (b) 5-membered heterocycles with one or two N substitutions. (c) Heterocycles with one or two amide groups. (d) Structures bearing 5- and 6-membered heterocyclic rings and quaternary N.

**
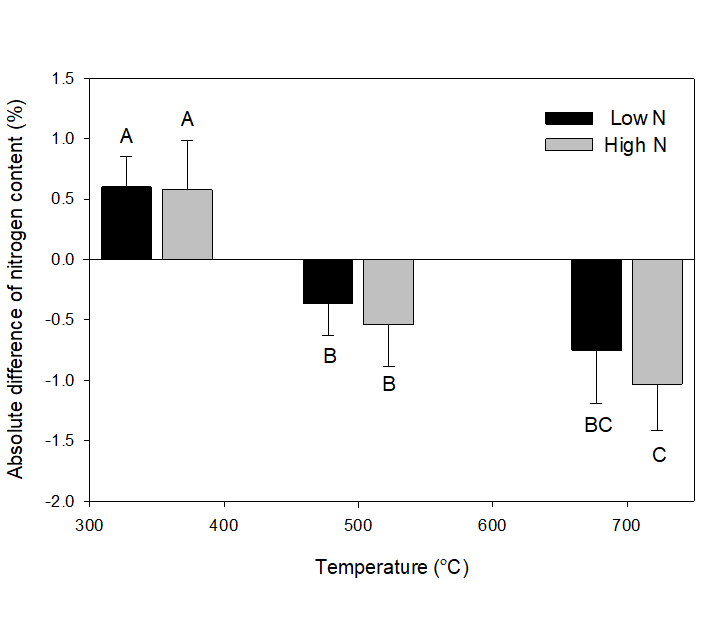
**

**Supplementary Figure 4.** Nitrogen enrichment or loss as a function of N content and temperature for all types of PyOM. Means and standard deviation (n=5). Different capital letters indicate significant differences (p<0.05) between treatments.

**
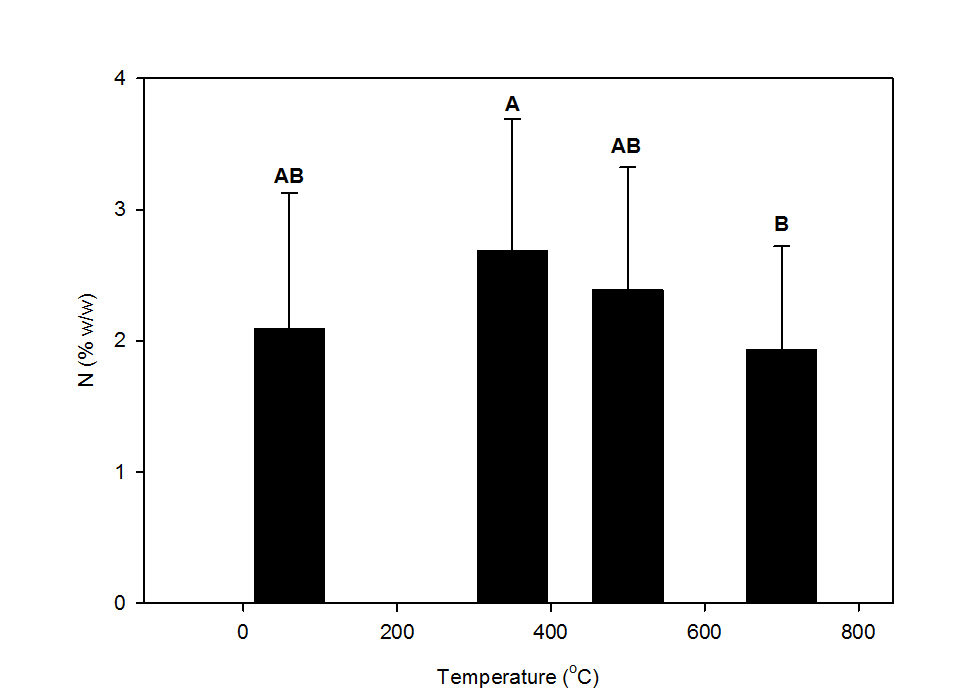
**

**Supplementary Figure 6.** Nitrogen contents as a function of temperature for uncharred OM and PyOM for all types of studied biomass. Means and standard deviation (n=14 except for uncharred OM n=15). Different capital letters indicate significant differences (p<0.05) between treatments.

**
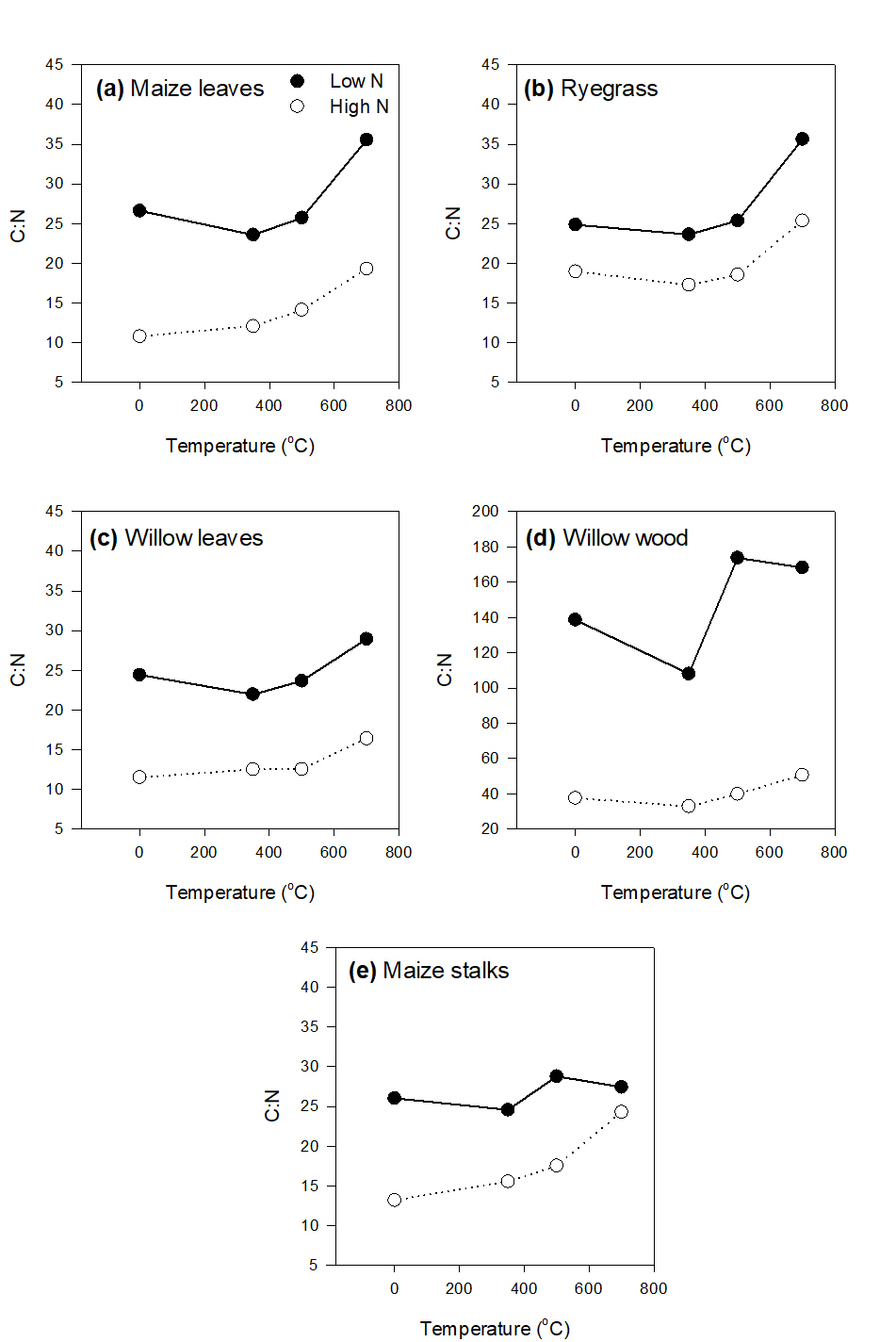
**

**Supplementary Figure 6.** C:N ratios as a function of pyrolysis temperature for uncharred OM and PyOM of (a) maize leaves, (b) ryegrass, (c) willow leaves, (d) willow wood and (e) maize stalks, for three pyrolysis temperatures. Black and white circles represent high- and low-N content of both OM and PyOM.

**
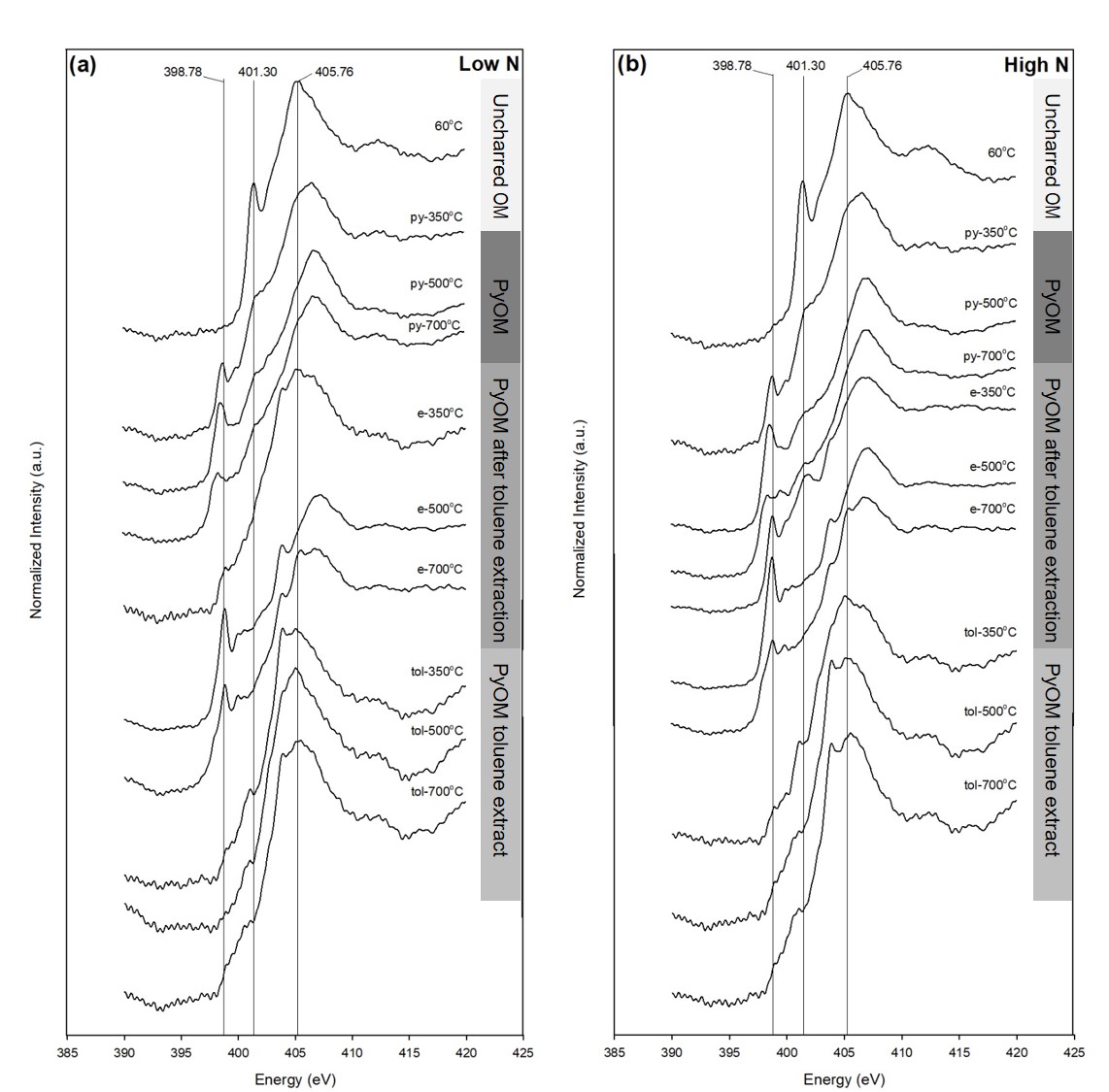
**

**Supplementary Figure 7.** **Nitrogen K-edge NEXAFS spectra of uncharred initial OM, entire PyOM (py-), extracted PyOM (e-), and the toluene extract of PyOM (tol-) as a function of pyrolysis temperature for willow leaves.** (a) High N-containing willow leaves, and (b) low N-containing willow leaves. Black lines represent the peak centers associated with selected key spectral features: 398.78 eV for C=N bonds in aromatic 6-membered rings, 401.30 eV for C-N bonds in aromatic 5-membered rings, and 405.76 eV N-H bonds.


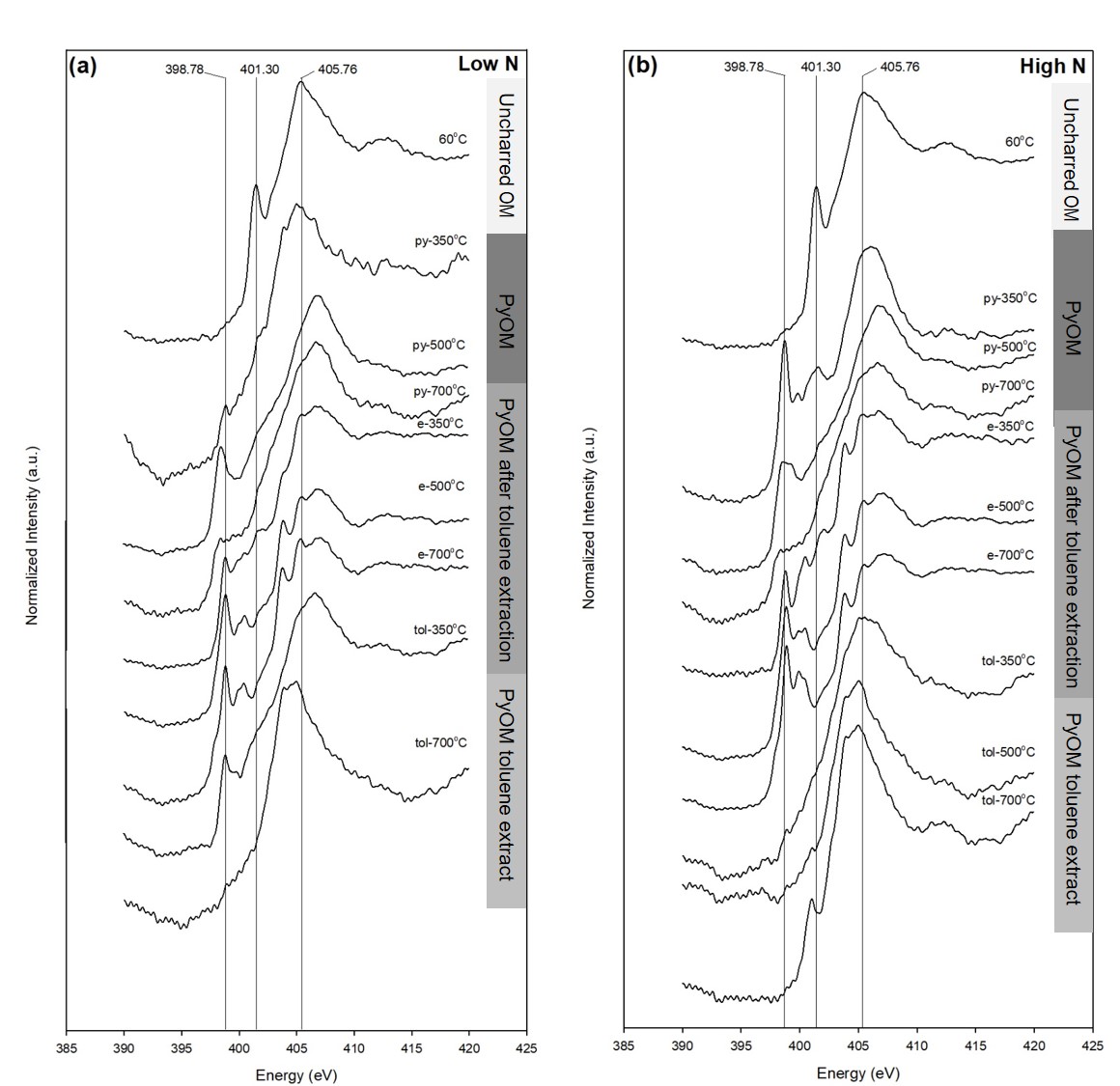


**Supplementary Figure 8.** **Nitrogen K-edge NEXAFS spectra of uncharred initial OM, entire PyOM (py-), extracted PyOM (e-), and the toluene extract of PyOM (tol-) as a function of pyrolysis temperature for maize stalks residues.** (a) Low N-containing maize stalks residues, and (b) high N-containing maize stalks residues. Black lines represent the peak centers associated with selected key spectral features: 398.78 eV for C=N bonds in aromatic 6-membered rings, 401.30 eV for C-N bonds in aromatic 5-membered rings, and 405.76 eV N-H bonds.


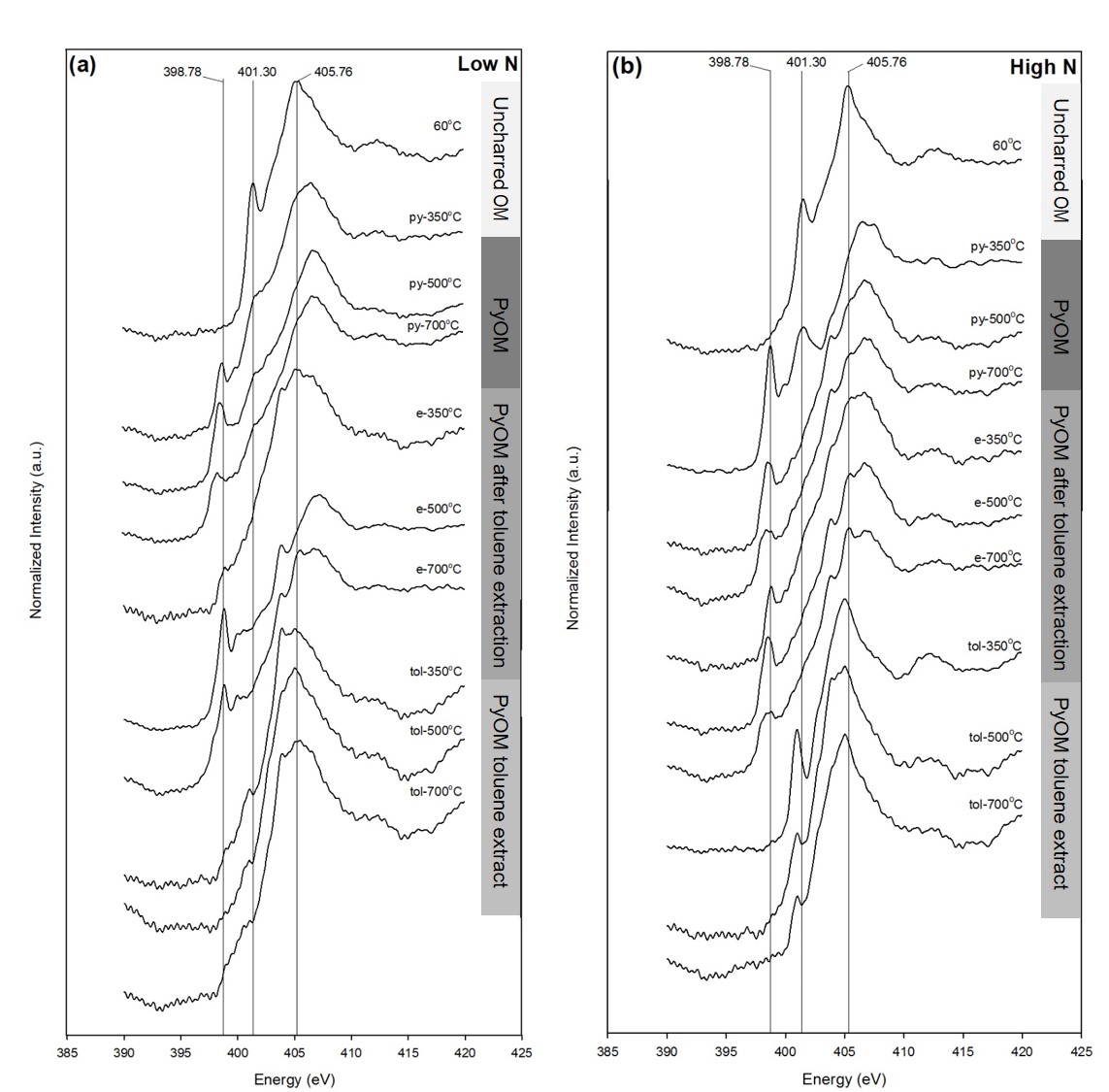


**Supplementary Figure 9.** **Nitrogen K-edge NEXAFS spectra of uncharred initial OM, entire PyOM (py-), extracted PyOM (e-), and the toluene extract of PyOM (tol-) as a function of pyrolysis temperature for willow wood residues.** (a) Low N-containing willow wood residues, and (b) high N-containing willow wood residues. Black lines represent the peak centers associated with selected key spectral features: 398.78 eV for C=N bonds in aromatic 6-membered rings, 401.30 eV for C-N bonds in aromatic 5-membered rings, and 405.76 eV N-H bonds.


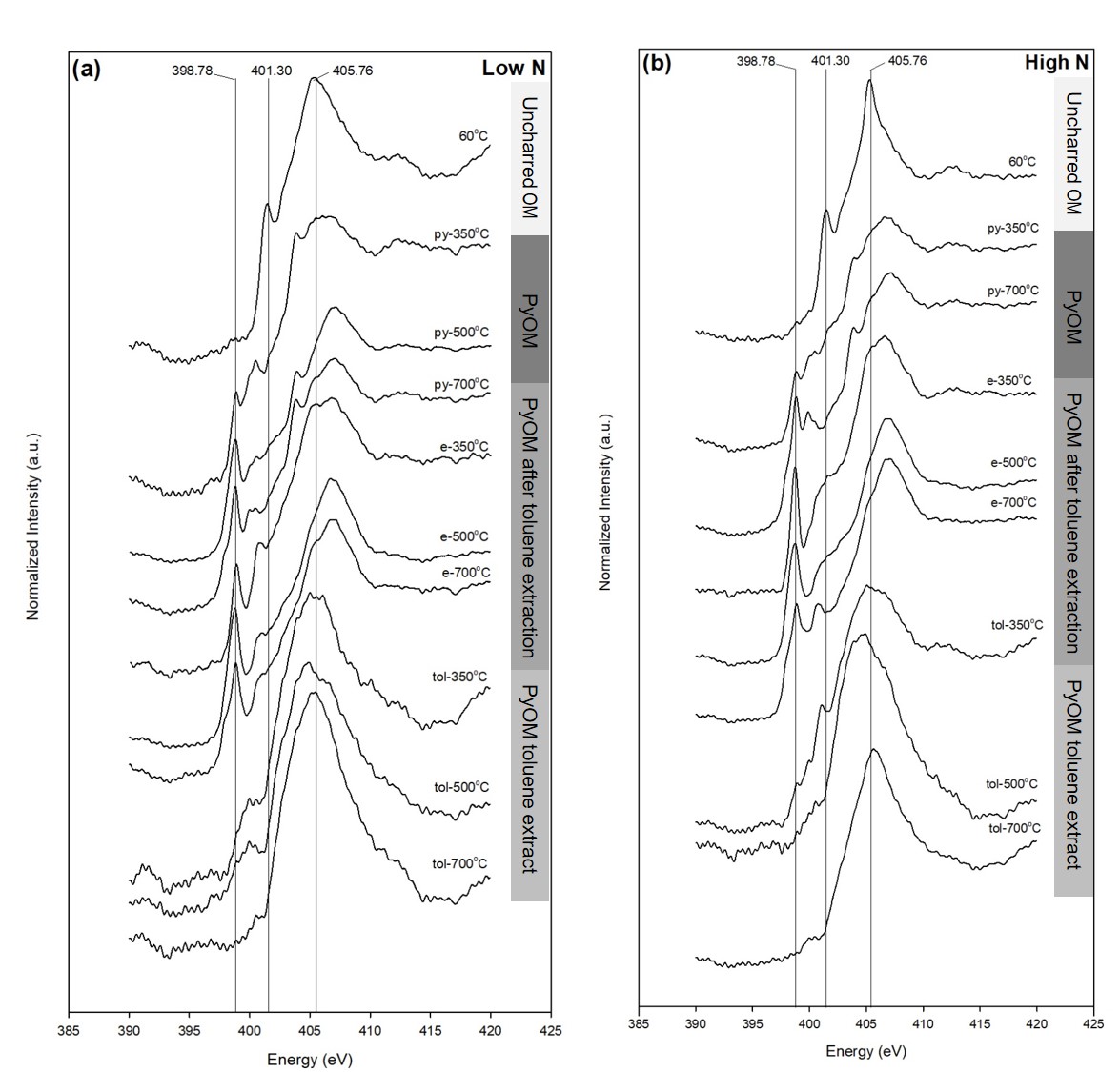


**Supplementary Figure 10.** **Nitrogen K-edge NEXAFS spectra of uncharred initial OM, entire PyOM (py-), extracted PyOM (e-), and the toluene extract of PyOM (tol-) as a function of pyrolysis temperature for ryegrass residues.** (a) Low N-containing ryegrass residues, and (b) high N-containing ryegrass residues. Black lines represent the peak centers associated with selected key spectral features: 398.78 eV for C=N bonds in aromatic 6-membered rings, 401.30 eV for C-N bonds in aromatic 5-membered rings, and 405.76 eV N-H bonds.


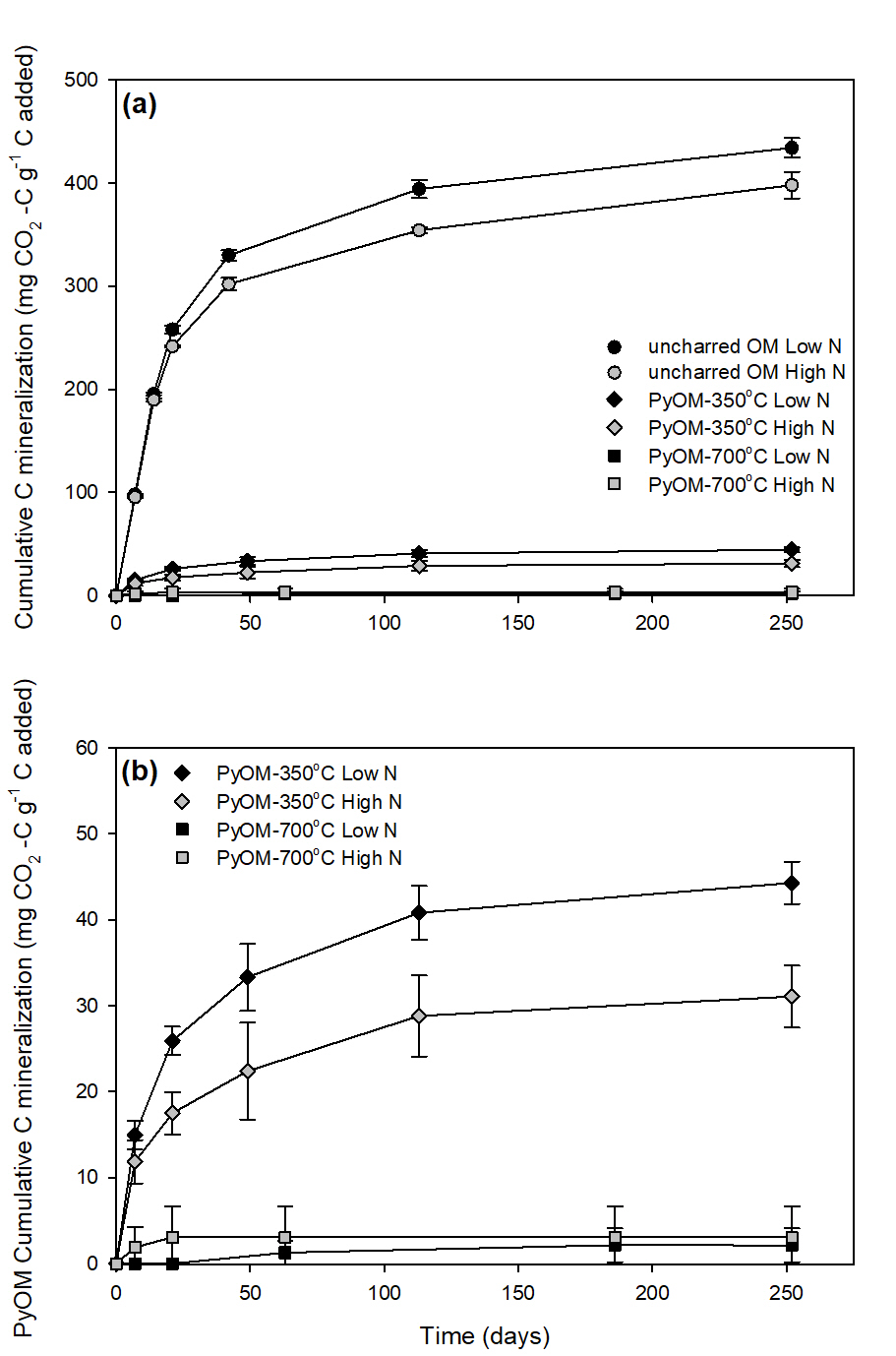


**Supplementary Figure 11.** **Cumulative C mineralized per unit C of added** **rye grass residues.** (a) Cumulative C mineralization for rye grass residues dried at 60°C and PyOM pyrolyzed at 350°C and 700°C at low and high N contents, and (b) cumulative C mineralization for PyOM at 350°C and 700°C only. Black circles, diamonds and squares represent low N-containing rye grass residues, whereas grey circles, diamonds and squares represent high N-containing rye grass residues.


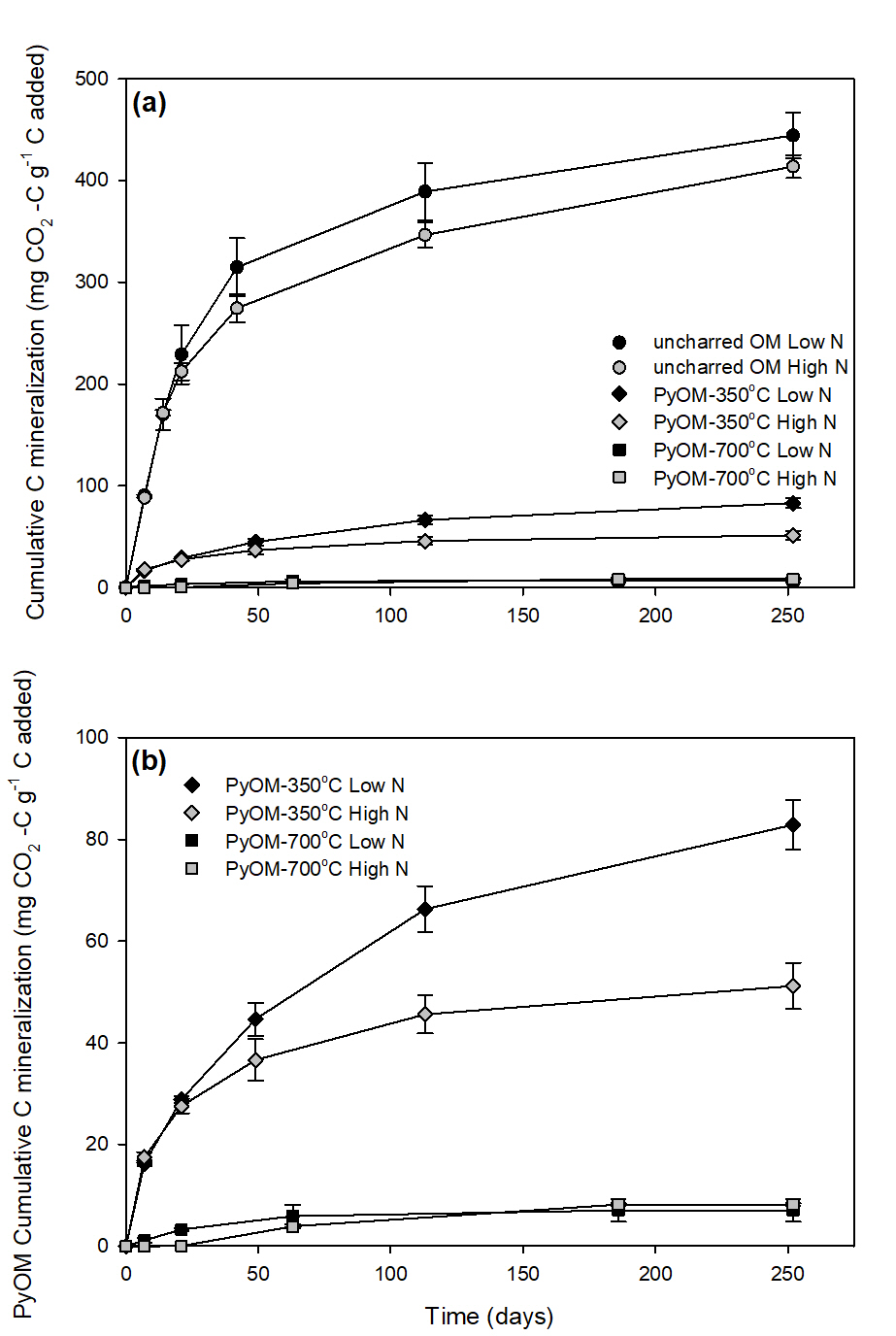


**Supplementary Figure 12.** **Cumulative C mineralized per unit C of added willow leaves.** (a) Cumulative C mineralization over time for willow leaves dried at 60°C and PyOM pyrolyzed at 350°C and 700°C, and (b) cumulative C mineralization for PyOM at 350°C and 700°C. Black circles, diamonds and squares represent low N-content willow leaves, whereas grey circles, diamonds and squares represent high N-containing willow leaves.


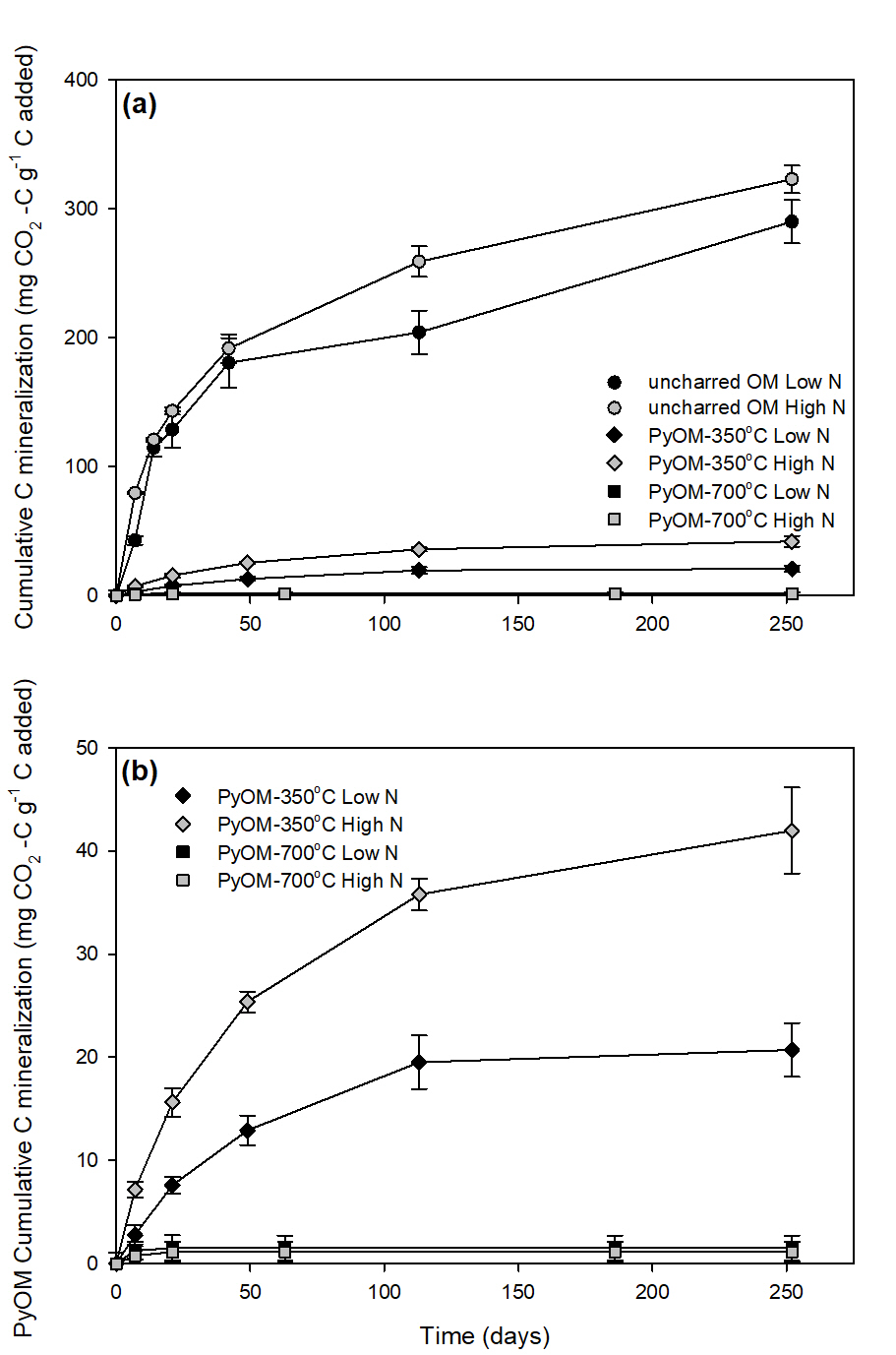


**Supplementary Figure 13.** **Cumulative C mineralized per unit C of added** **willow wood residues.** (a) Cumulative C mineralization over time for willow wood residues dried at 60°C and PyOM pyrolyzed at 350°C and 700°C, and (b) cumulative C mineralization for PyOM at 350°C and 700°C. Black circles, diamonds and squares represent low N-containing willow wood residues, whereas grey circles, diamonds and squares represent high N-containing willow wood residues.

**
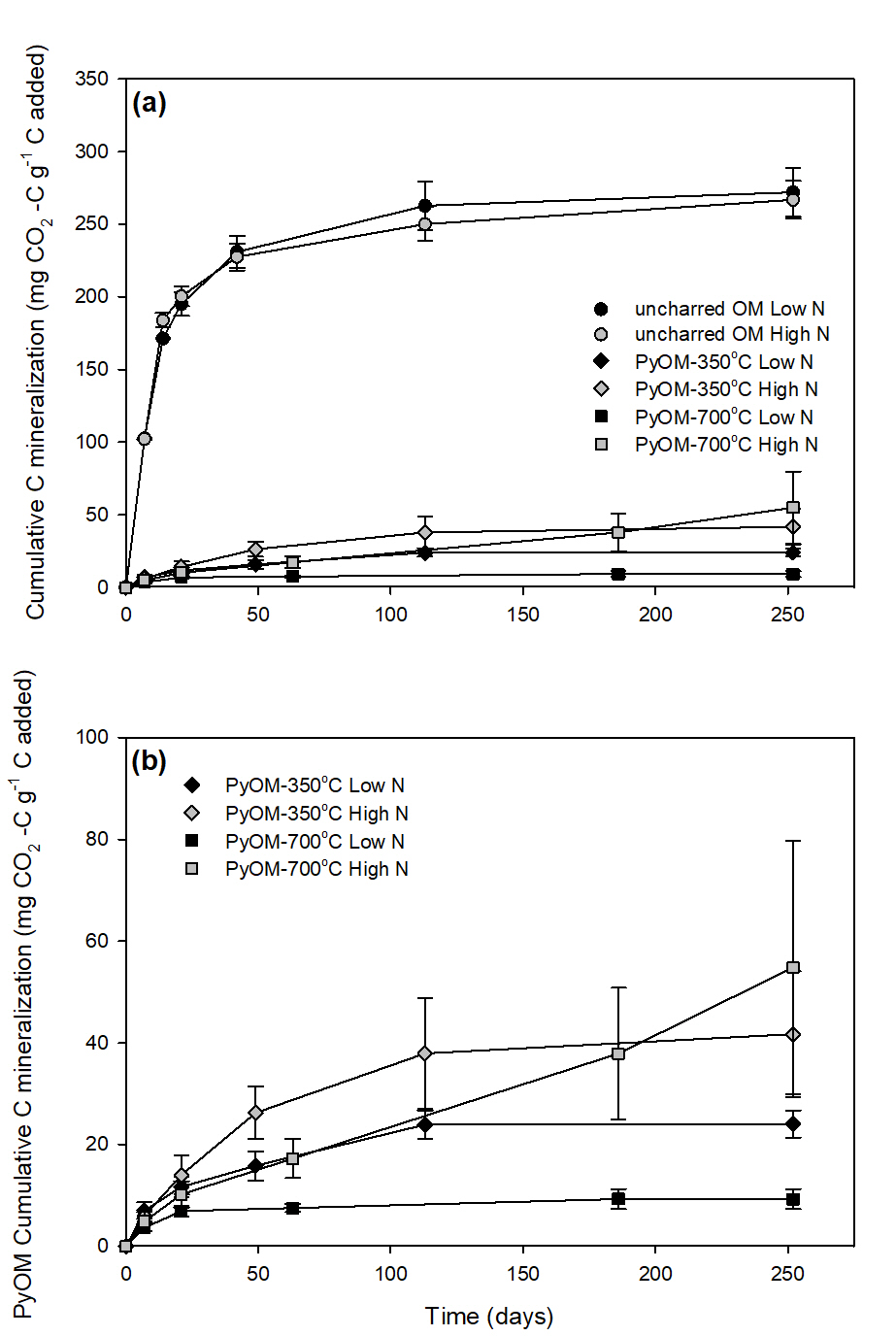
**

**Supplementary Figure 14.** **Cumulative C mineralized per unit C of added** **maize stover.** (a) Cumulative C mineralization over time for maize stover dried at 60°C and PyOM pyrolyzed at 350°C and 700°C, and (b) cumulative C mineralization for PyOM at 350°C and 700°C. Black circles, diamonds and squares represent low N-containing maize stalks, whereas grey circles, diamonds and squares represent high N-containing maize stalks.


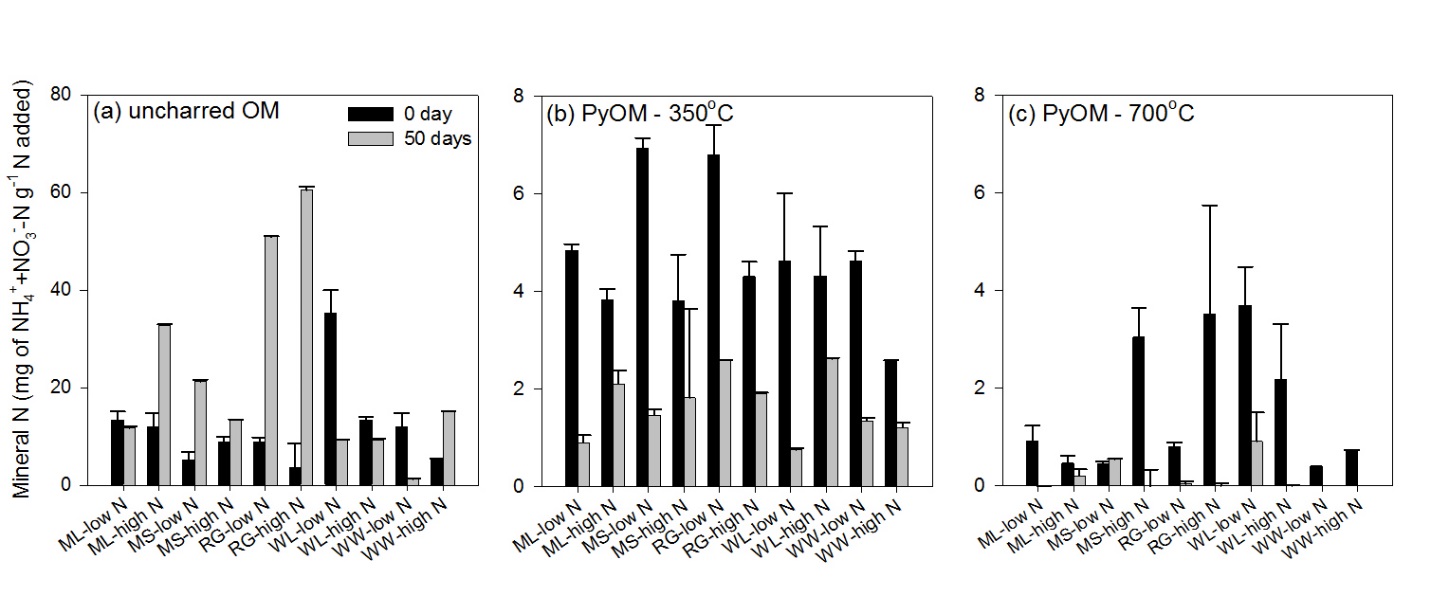


**Supplementary Figure 15. Extracted mineral N in the form of NH_4_^+^and NO_3_^-^**. (a) Mineral N over time for OM with low and high N content, (b) mineral N over time for PyOM produced at 350°C with low and high N content, and (c) mineral N over time for PyOM produced at 700°C with low and high N content. Black vertical bars represent extractable mineral N (NH_4_^+^ and NO_3_^-^) before the incubation started, whereas grey vertical bars represent total extractable mineral N at day 50 of the incubation. The bars are an average of duplicates for day 0 and 4 replicates at day 50 (ML: maize leaves, MS: maize stover, RG: Ryegrass, WL: willow leaves, WW: willow wood).


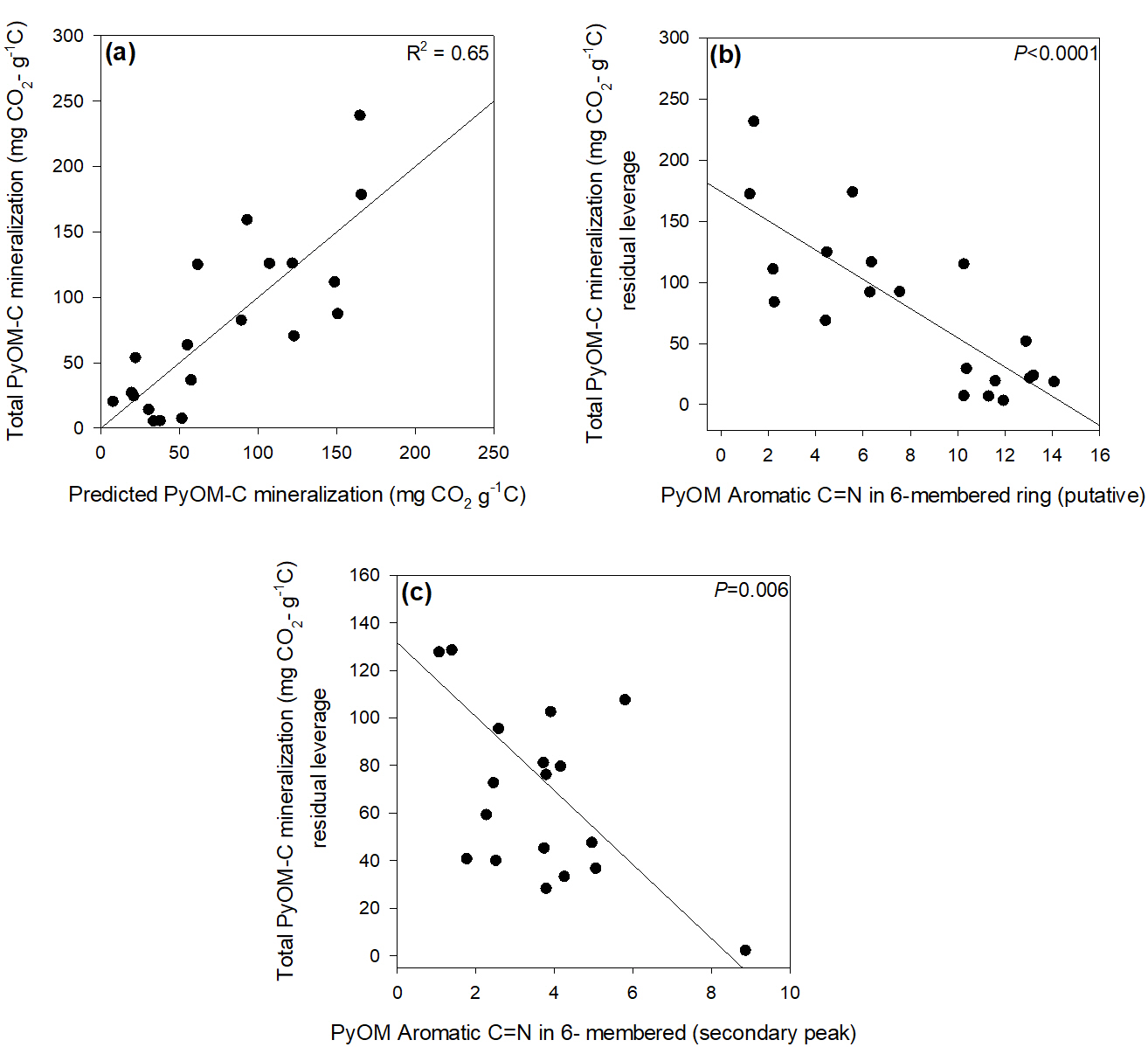


**Supplementary Figure 16. Relationship between cumulative PyOM-C mineralized and ratios of N functional groups from PyOM identified by N K-edge XANES.** (a) Actual vs. predicted response values using a multiple regression model for cumulative PyOM-C mineralized, (b) relationship between cumulative PyOM-C mineralized and aromatic C=N in 6-membered rings (putative) from PyOM, and (c) relationship between cumulative PyOM-C mineralized and aromatic C=N in 6-membered rings (secondary peak).


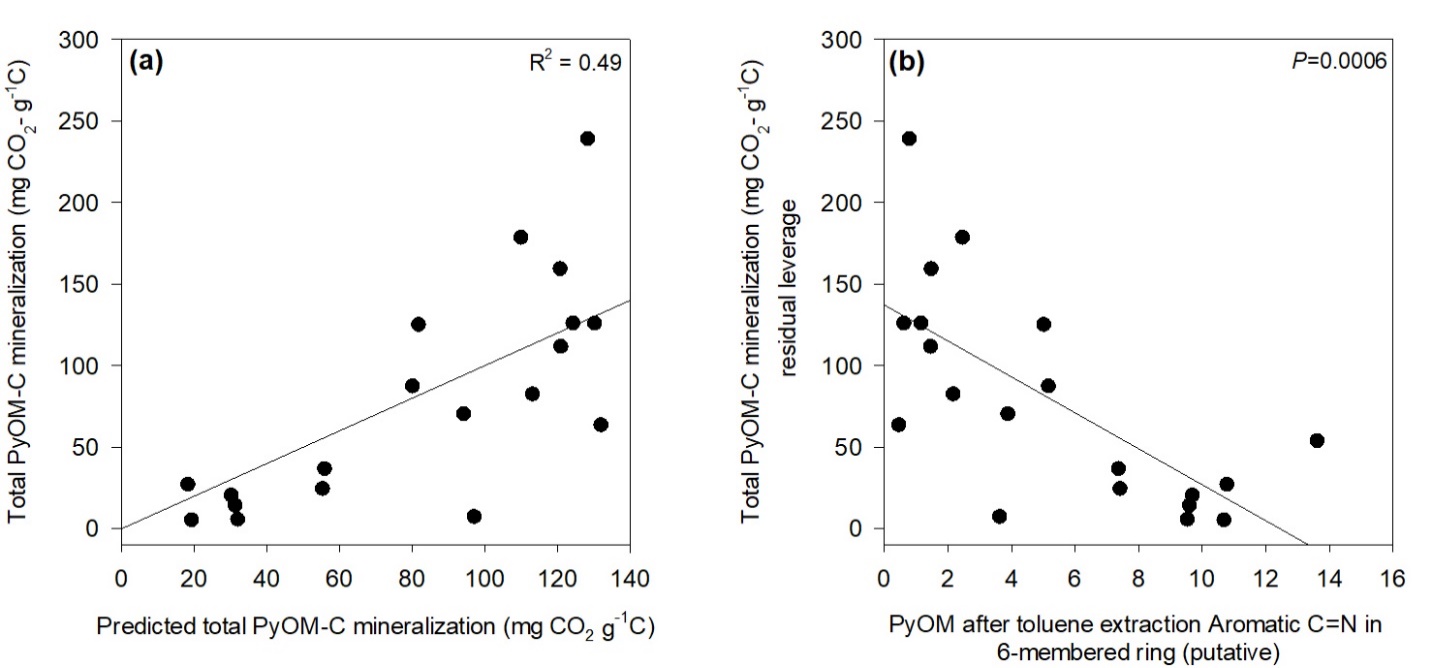


**Supplementary Figure 17. Relationship between cumulative PyOM-C mineralized and ratios of N functional groups from PyOM after toluene extraction identified by N K-edge XANES.** (a) Actual vs. predicted response values using a multiple regression model for cumulative PyOM-C mineralized, and (b) relationship between cumulative PyOM-C mineralized and aromatic C=N in 6-membered rings (putative) from PyOM after toluene extraction.

**Supplementary Table 1**

Elemental composition for OM and PyOM of all feedstocks.

|  |  | **Treatment** | | | | | |
| --- | --- | --- | --- | --- | --- | --- | --- |
|  |  | **Low N** | | | **High N** | | |
| **Feedstock** | **T (°C)** | **Total C**  **(mg g^-1^)** | **Total N**  **(mg g^-1^)** | **C/N** | **Total C**  **(mg g^-1^)** | **Total N**  **(mg g^-1^)** | **C/N** |
| **Maize leaves** | 60 | 421.37 | 15.83 | 26.61 | 426.85 | 39.43 | 10.82 |
|  | 350 | 539.65 | 22.90 | 23.57 | 510.80 | 42.35 | 12.06 |
|  | 500 | 555.50 | 21.60 | 25.72 | 501.55 | 35.55 | 14.11 |
|  | 700 | 540.20 | 15.20 | 35.54 | 501.40 | 25.95 | 19.32 |
| **Maize stalks** | 60 | 381.40 | 14.70 | 25.95 | 380.80 | 29.00 | 13.13 |
|  | 350 | 465.40 | 18.95 | 24.56 | 454.45 | 29.25 | 15.54 |
|  | 500 | 569.55 | 20.30 | 28.06 | 407.10 | 23.25 | 17.51 |
|  | 700 | 480.70 | 23.70 | 20.28 | 460.15 | 18.90 | 24.35 |
| **Willow leaves** | 60 | 421.20 | 17.30 | 24.35 | 440.20 | 38.30 | 11.49 |
|  | 350 | 553.10 | 25.20 | 21.95 | 561.30 | 44.95 | 12.49 |
|  | 500 | 541.60 | 22.90 | 23.65 | 437.90 | 34.90 | 12.55 |
|  | 700 | 577.40 | 19.95 | 28.94 | 587.60 | 35.85 | 16.39 |
| **Willow wood** | 60 | 450.80 | 3.30 | 136.61 | 446.30 | 11.90 | 37.50 |
|  | 350 | 707.20 | 6.55 | 107.97 | 682.80 | 20.85 | 32.75 |
|  | 500 | 765.05 | 4.40 | 173.88 | 749.85 | 18.85 | 39.78 |
|  | 700 | 808.00 | 4.80 | 168.33 | 755.05 | 14.95 | 50.51 |
| **Ryegrass** | 60 | 397.50 | 16.00 | 24.84 | 409.50 | 21.60 | 18.96 |
|  | 350 | 503.05 | 21.30 | 23.62 | 543.55 | 31.50 | 17.26 |
|  | 500 | 515.00 | 20.30 | 25.37 | 547.00 | 29.50 | 18.54 |
|  | 700 | 461.25 | 12.95 | 35.62 | 551.90 | 21.75 | 25.37 |

**Supplementary Table 2**

pH and inorganic NH_4_-N and NO_3_-N concentrations for OM and PyOM of all feedstocks.

|  |  | **Treatment** | | | | | |
| --- | --- | --- | --- | --- | --- | --- | --- |
|  |  | **Low N** | | | **High N** | | |
| **Feedstock** | **T (°C)** | **NH_4_-N (mg g^-1^)** | **NO_3_-N (mg g^-1^)** | **pH** | **NH_4_-N (mg g^-1^)** | **NO_3_-N**  **(mg g^-1^)** | **pH** |
| **Maize leaves** | 60 | 9.42 | 0.33 | 5.04 | 4.60 | 1.33 | 9.42 |
|  | 350 | 4.80 | 0.05 | 6.92 | 3.58 | 0.26 | 8.40 |
|  | 700 | 0.79 | 0.12 | 9.42 | 0.44 | 0.02 | 9.40 |
| **Maize stalks** | 60 | 13.62 | 0.50 | 5.86 | 21.28 | 8.91 | 6.00 |
|  | 350 | 6.83 | 0.11 | 7.12 | 3.64 | 0.16 | 8.40 |
|  | 700 | 0.45 | 0.01 | 9.31 | 2.57 | 0.47 | 9.45 |
| **Willow leaves** | 60 | 11.78 | 0.24 | 5.23 | 4.80 | 0.38 | 5.32 |
|  | 350 | 4.39 | 0.24 | 7.16 | 6.96 | 0.16 | 7.62 |
|  | 700 | 0.40 | 0.00 | 9.85 | 0.70 | 0.00 | 10.12 |
| **Willow wood** | 60 | 33.31 | 2.02 | 5.18 | 12.68 | 0.67 | 5.33 |
|  | 350 | 4.39 | 0.24 | 6.08 | 6.96 | 0.16 | 6.64 |
|  | 700 | 2.63 | 1.06 | 8.93 | 1.81 | 0.38 | 9.19 |
| **Ryegrass** | 60 | 8.94 | 0.00 | 6.11 | 3.47 | 0.12 | 5.91 |
|  | 350 | 6.73 | 0.08 | 7.07 | 4.25 | 0.05 | 7.07 |
|  | 700 | 0.66 | 0.13 | 9.82 | 3.37 | 0.14 | 9.57 |

**Supplementary Table 3**

Major features of TEY N K-edge NEXAFS spectra collected from N standard compounds. Peak centers and half widths at half maximum (hwhm) are shown in eV.

|  |  | | | | | | | | | |
| --- | --- | --- | --- | --- | --- | --- | --- | --- | --- | --- |
| **Standard compound** | **1*s*→π*** | **hwhm** | **1*s*→π*** | **hwhm** | **1*s*→π*** | **hwhm** | **1*s*→σ*** | **hwhm** | **1s→σ*** | **hwhm** |
| Acridine | 398.10 | 0.42 |  |  |  |  | 406.98 | 2.41 |  |  |
| 2,3-Pyrazinedicaboxylic acid | 398.72 | 0.30 | 400.25 | 0.41 | 402.48 | 0.37 | 407.50 | 1.48 |  |  |
| 2,3-Pyridinedicaboxylic acid | 398.89 | 0.33 | 400.04 | 0.61 | 402.47 | 1.60 | 403.88 | 0.47 | 408.12 | 1.24 |
| 2-Pyrimidinecarbonitrile | 398.80 | 0.40 | 400.00 | 0.40 | 402.60 | 0.40 | 404.50 | 2.00 | 408.60 | 2.00 |
| Cytosine | 399.20 | 0.40 | 400.60 | 0.40 | 403.00 | 0.40 | 405.00 | 2.00 | 408.40 | 2.00 |
| 3,5-Pyrazoledicarboxylic acid | 399.90 | 0.41 | 401.18 | 0.68 |  |  | 406.04 | 1.70 | 410.37 | 2.38 |
| L-Histidine | 399.97 | 0.40 | 401.41 | 0.51 |  |  | 406.25 | 1.95 | 412.82 | 3.06 |
| 4-Nitro-1H-pyrazole | 400.35 | 0.40 | 401.02 | 0.50 | 403.79 | 0.37 | 412.77 | 3.77 |  |  |
| 1-Ethyl-4-(methoxycarbonyl)-pyridinium iodide | 400.49 | 0.36 |  |  | 404.04 | 0.36 | 408.36 | 2.05 |  |  |
| 2-Pyridone | 400.74 | 0.54 |  |  |  |  | 406.92 | 2.05 |  |  |
| Glycine anhydride | 401.12 | 0.42 |  |  |  |  | 405.39 | 1.76 | 412.40 | 2.39 |
| Carbazole | 402.13 | 0.37 |  |  |  |  | 407.19 | 1.87 |  |  |
| 4-Nitro-phenylacetic acid | 403.44 | 0.43 |  |  |  |  | 411.78 | 2.48 |  |  |
| Ammonium sulfate |  |  |  |  |  |  | 405.76 | 1.37 |  |  |
| L-Isoleucine |  |  |  |  |  |  | 406.10 | 1.83 |  |  |

**Supplementary Table 4.**

N K-edge NEXAFS TEY peak assignments used in deconvolution model.

| **N group** | | **Bond** | | **Peak Energy (eV) 1s→π*** | **Fit position** | **Peak Energy (eV) 1s→σ*** |
| --- | --- | --- | --- | --- | --- | --- |
| Aromatic C=N in 6-membered ring (putative) | | | C=N | 398.10 | 398.10 |  |
| Aromatic C=N in 6-membered ring (1&2N) | | | C=N | 398.70-398.89 | 398.78 | 407.80 |
| Aromatic C=N in 6-membered oxygenated ring (2N) | | | C=N | 399.40 | 399.40 |  |
| Aromatic C=N in 5-membered ring (2N) | | | C=N | 399.90-399.97 | 399.93 | 406.14, 410.37, 412.82 |
| Nitrile | | | C≡N | 400.00 | 400.00 | 404.50, 408.60 |
| Quaternary C-N in 6-membered ring | | | C-N | 400.49 | 400.49 | 408.36 |
| Amide N in an aromatic 6-membered ring (2N) | O=C-N | | | 400.74 | 400.74 | 406.92 |
| Amide N in a non-aromatic 6-membered ring (2N) | | | O=C-N | 401.12 | 401.12 | 405.39,412.4 |
| Amide N* or Aromatic C-N in 5-membered ring (2N) | | | O=C-N/C-N | 401.18-401.41 | 401.30 | 406.14,410.37,412.82 |
| Aromatic C-N in 5-membered ring (1N) | | | C-N | 402.13 | 402.13 | 407.19 |
| Aromatic C=N in 6-membered ring (1&2N, secondary peak) | | | C=N | 402.48-402.60 | 402.54 | 408.05 |
| Aliphatic C-NH_2_ bonded to aromatic 6-membered ring | | | C-NH_2_ | 403.00 | 403.00 |  |
| Nitro C-NO_2_ bonded to aromatic 6-membered ring | | | C-NO_2_ | 403.44-403.79 | 403.61 | 412.28 |
| Aromatic N secondary peak | | | C=N | 403.86-404.04 | 403.95 | 408.24 |
| Inorganic N-H | | | N-H |  |  | 405.76 |
| Aliphatic C-NH_2_ | | | C-NH_2_ |  |  | 406.10 |

Median values were calculated for N structures represented in more than one standard compound, which constituted the designated fit position used in the final model. The peak centered at 398.10 eV is not associated with a specific standard compound, but was identified by deconvolution of PyOM spectra and is closest in position to spectral features associated with aromatic 6-membered rings.

^*^ The peak at 401.30 eV is assigned to multiple N groups due to a high degree of energy position overlap between the 1s→π* of amides and the resonance of the delocalized pair of electrons in 5-membered aromatic heterocycles.

**Supplementary Table 5.**

Proportion of total N as a fraction of PyOM-N for varying types of OM and different levels of N contents.

| **Proportion of total N as a fraction of PyOM** | | | | | | |
| --- | --- | --- | --- | --- | --- | --- |
|  | **Low N** | | | | | |
| **Feedstock** | **py-350** | **e-350** | **tol-350** | **py-700** | **e-700** | **tol-700** |
| ML | 1 | 0.71 | 0.29 | 1 | 0.84 | 0.16 |
| MS | 1 | 0.69 | 0.31 | 1 | 0.65 | 0.35 |
| RG | 1 | 0.64 | 0.36 | 1 | 0.98 | 0.02 |
| WL | 1 | 0.52 | 0.48 | 1 | 0.66 | 0.34 |
| WT | 1 | 0.84 | 0.16 | 1 | 0.82 | 0.18 |
|  | **High N** | | | | | |
|  | **py-350** | **e-350** | **tol-350** | **py-700** | **e-700** | **tol-700** |
| ML | 1 | 0.89 | 0.11 | 1 | 0.98 | 0.02 |
| MS | 1 | 0.34 | 0.66 | 1 | 0.54 | 0.46 |
| RG | 1 | 0.45 | 0.55 | 1 | 0.89 | 0.11 |
| WL | 1 | 0.72 | 0.28 | 1 | 0.81 | 0.19 |
| WT | 1 | 0.93 | 0.07 | 1 | 0.96 | 0.04 |

Nitrogen mass balance between PyOM (py-), PyOM after toluene extraction (e-) and PyOM toluene extract (tol-) of maize leaves (ML), maize stalk (MS), ryegrass (RG), willow leaves (WL) and willow wood (WT).

**Supplementary Table 6**

Proportion of absorption area (%) of N bonds measured with N K-edge NEXAFS for maize leaves OM and PyOM components (as a fraction of the sum of all resolved N functional groups).


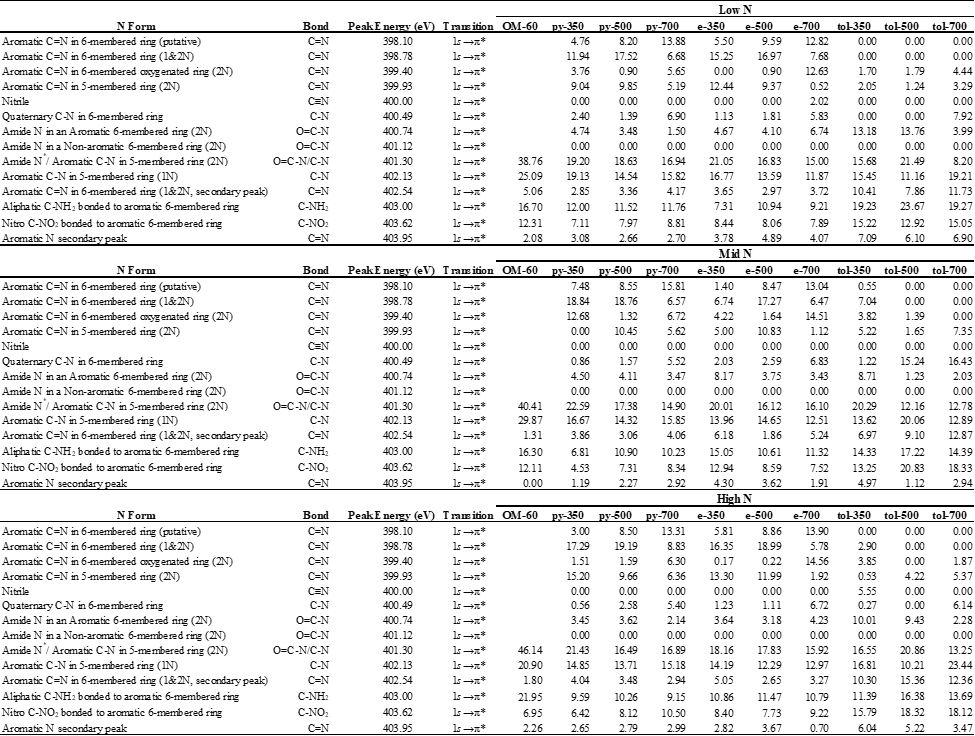


Deconvolution of NEXAFS spectra collected from OM, PyOM, e-PyOM and tol-PyOM. PyOM was used to determine the π area represented by different N bonds. Proportions of areas were only calculated for π bonds because of the high degree of peak overlap in the σ region.

**Supplementary Table 7**

Proportion of absorption regions (%) of N bonds measured with N K-edge NEXAFS for maize stover OM and PyOM components (as a fraction of the sum of all resolved N functional groups).

**Supplementary Table 8**

Proportion of absorption regions (%) of N bonds measured with N K-edge NEXAFS for ryegrass OM and PyOM components (as a fraction of the sum of all resolved N functional groups).

**Supplementary Table 9**

Proportion of absorption regions (%) of N bonds measured with N K-edge NEXAFS for willow wood OM and PyOM components (as a fraction of the sum of all resolved N functional groups).

**Supplementary Table 10**

Proportion of absorption regions (%) of N bonds measured with N K-edge NEXAFS for willow leaves OM and PyOM components (as a fraction of the sum of all resolved N functional groups).

**References**

Cheng C.-H., Lehmann J., Thies J. E. and Burton S. D. (2008) Stability of black carbon in soils across climatic gradient. *J. Geophys. Res. Biogeosciences* **113**, G02027.

Le Guillou C., Bernard S., De la Pena F. and Le Brech Y. (2018) XANES-based quantification of carbon functional group concentrations. *Anal. Chem.* **90**, 8379–8386.
